# Supplementary material for: Genomic Regions and Candidate Genes Affecting Response to Heat Stress with Newcastle Virus Infection in Commercial Layer Chicks Using Chicken 600K Single Nucleotide Polymorphism Array
Source: Int J Mol Sci. 2024 Feb 24;25(5):2640. doi: 10.3390/ijms25052640 (PMC10931805; doi:10.3390/ijms25052640)
Supplement: Supplementary file 1 [file ijms-25-02640-s001.zip › Supplementary file S1 Lease square means and P-values of blood parameters with treatments.pdf]

Table S1. Lease square means and *P-values* of blood parameters with treatments.

		LS Mean ± SE		P-values		
	**Blood Parameter**	**Control**	**Treated**	**Trt**	**Sex**	**Trt*Sex**
**Acute Heat (AH)**	Na^+ (mmol/L)	129.62±0.91	129.44±0.27	0.6205	0.9369	0.1287
	K^+ (mmol/L)	4.32±0.07	4.29±0.02	0.7719	0.2594	0.13
	iCa^{2+} (mmol/L)	0.45±0.07	0.76±0.03	0.5531	0.7541	0.339
	Glu (mg/dL)	241.72±3.00	249.94±0.94	0.3633	0.6815	0.1691
	pH	7.46±0.009	7.42±0.003	0.1533	0.2874	0.9615
	PCO_2 (mmHg)	30.98±0.76	32.63±0.24	0.323	0.6564	0.6222
	TCO_2(mmol/L)	23.42±0.30	22.08±0.09	0.3774	0.9779	0.5105
	HCO_3 (mmol/L)	22.51±0.29	21.09±0.09	0.3765	0.8204	0.5843
	PO_2 (mmHg)	61.38±1.38	55.37±1.24	0.1144	0.8326	0.964
	sO_2 (%)	92.45±0.82	86.74±0.26	0.113	0.4692	0.3884
	BE (mmol/L)	-1.23±0.35	-3.35±0.11	0.3846	0.5564	0.4883
**Chronic Heat (CH)**	Na^+ (mmol/L)	129.99±0.84	129.55±0.23	0.8913	0.0463	0.1875
	K^+ (mmol/L)	4.29±0.08	4.28±0.02	0.9095	0.8872	0.4046
	iCa^{2+} (mmol/L)	0.87±0.03	0.84±0.01	0.5378	0.0702	0.308
	Glu (mg/dL)	240.50±3.57	239.63±0.95	0.7872	0.7735	0.5109
	pH	7.42±0.01	7.41±0.01	0.3451	0.3216	0.6793
	PCO_2 (mmHg)	32.49±0.38	30.79±0.24	0.4781	0.3671	0.3865
	TCO_2(mmol/L)	22.03±0.38	20.33±0.10	0.3635	0.4069	0.1356
	HCO_3 (mmol/L)	21.07±0.36	19.41±0.10	0.3462	0.4269	0.1494
	PO_2 (mmHg)	60.80±1.32	57.83±0.35	0.3025	0.4143	0.1295
	sO_2 (%)	91.12±0.72	89.31±0.19	0.2322	0.5876	0.165
	BE (mmol/L)	$-3.33±0.42^{A}$	$-5.20±0.11^{B}$	0.0005	0.8871	0.8147
**Chronic Heat 2 (CH&NDV)**	Na^+ (mmol/L)	114.25±1.45	127.00±0.38	0.4517	0.8089	0.0807
	K^+ (mmol/L)	3.35±0.09	4.40±0.02	0.277	0.2715	0.811
	iCa^{2+} (mmol/L)	0.32±0.05	0.89±0.01	0.359	0.9265	0.5744
	Glu (mg/dL)	$235.90±3.14^{B}$	$237.75±0.81^{A}$	0.0009	0.6531	0.1044
	pH	7.44±0.01	7.44±0.01	0.289	0.1823	0.7631
	PCO_2 (mmHg)	33.16±0.85	28.76±0.22	0.9838	0.5579	0.5682
	TCO_2(mmol/L)	23.15±0.41	20.17±0.10	0.0973	0.0168	0.1272
	HCO_3 (mmol/L)	22.18±0.39	19.32±0.10	0.0945	0.0116	0.1406
	PO_2 (mmHg)	61.00±1.30	55.90±0.34	0.6984	0.0059	0.0159
	sO_2 (%)	91.15±0.74	89.33±0.19	0.8616	0.6827	0.5621
	BE (mmol/L)	-1.94±0.43	-4.83±0.11	0.0988	0.0064	0.1878

Note: Control: non-treated group; Treated: treated group; Trt: treatment effects; Sex: Sex effects; Trt*Sex: interaction effects of treatment and sex; Different superscript letters within the row represent significant differences (P<0.05).
