# Supplementary material for: Genomic Regions and Candidate Genes Affecting Response to Heat Stress with Newcastle Virus Infection in Commercial Layer Chicks Using Chicken 600K Single Nucleotide Polymorphism Array
Source: Int J Mol Sci. 2024 Feb 24;25(5):2640. doi: 10.3390/ijms25052640 (PMC10931805; doi:10.3390/ijms25052640)
Supplement: Supplementary file 1 [file ijms-25-02640-s001.zip › Supplementary file S2 GWAS Manhattan plots.pptx]

## Slide 1
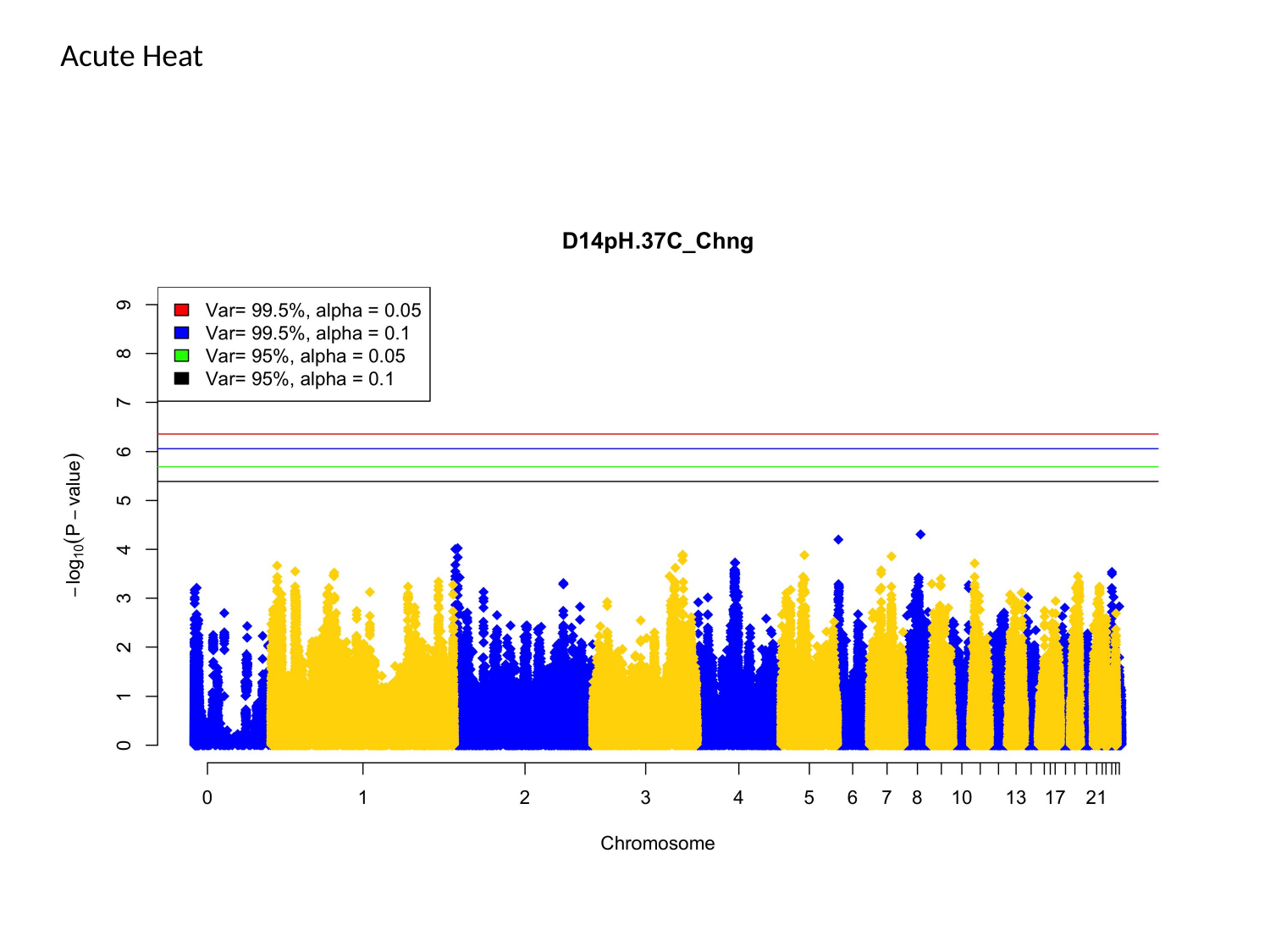

Acute Heat

## Slide 2
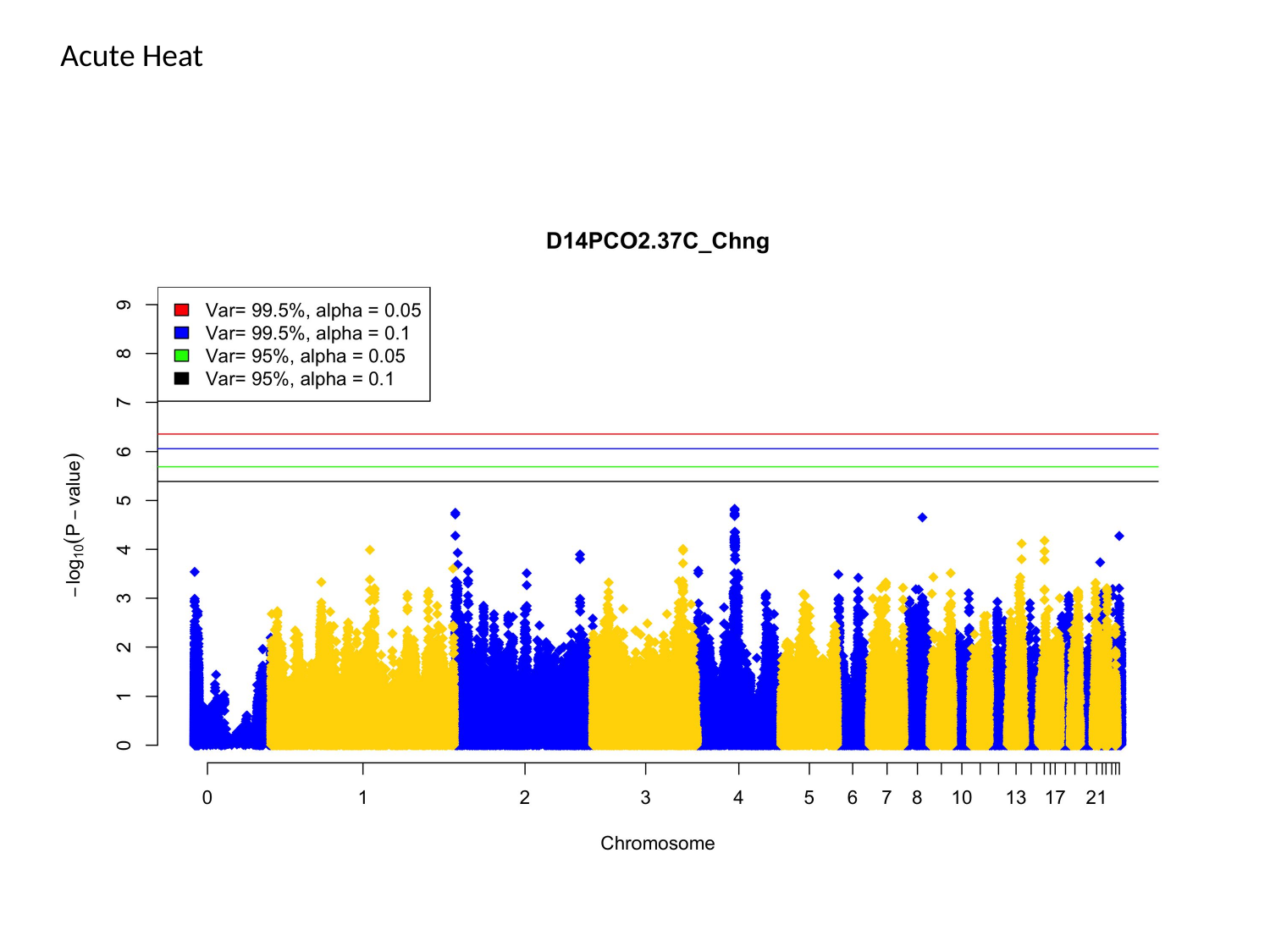

Acute Heat

## Slide 3
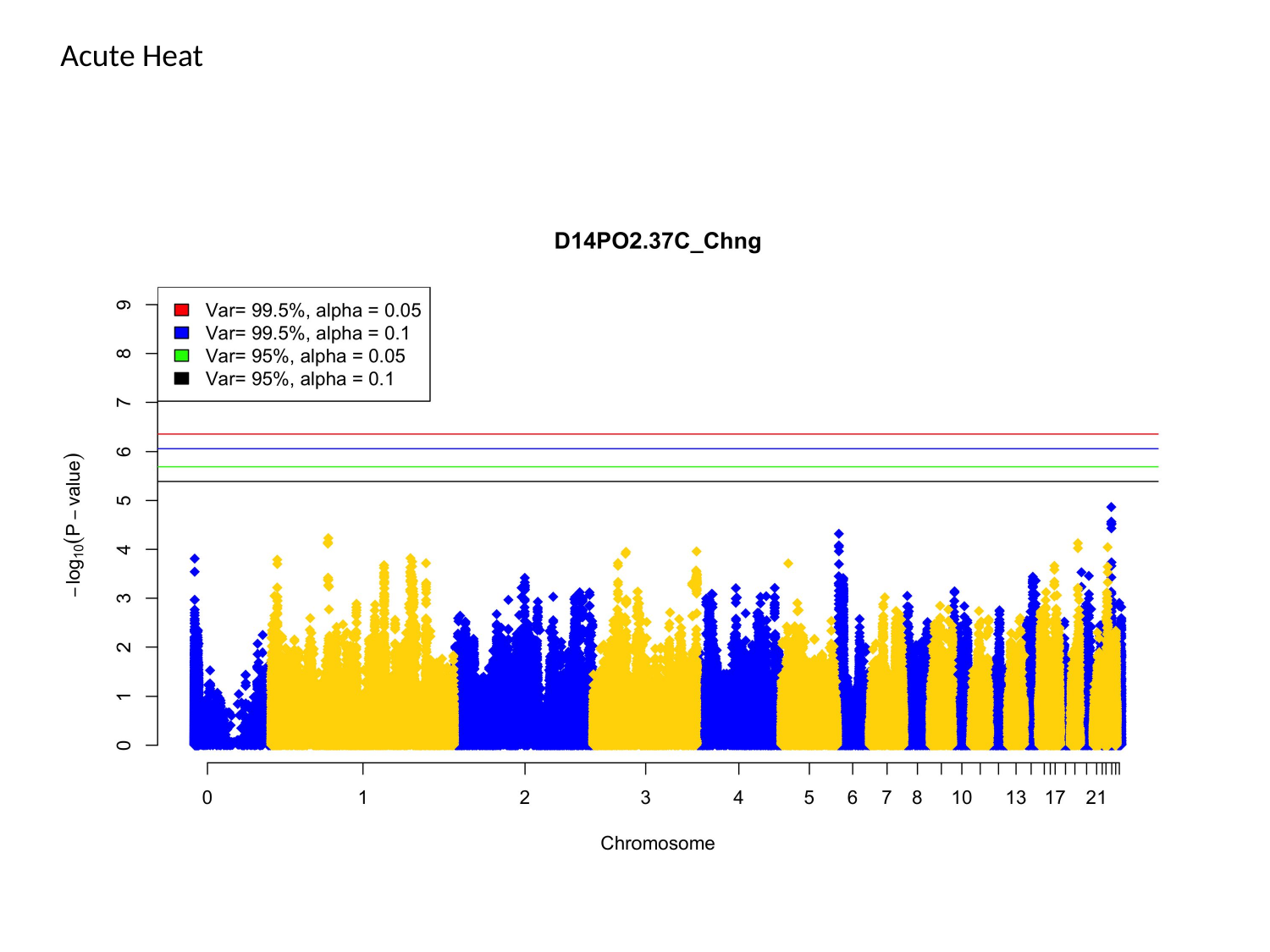

Acute Heat

## Slide 4
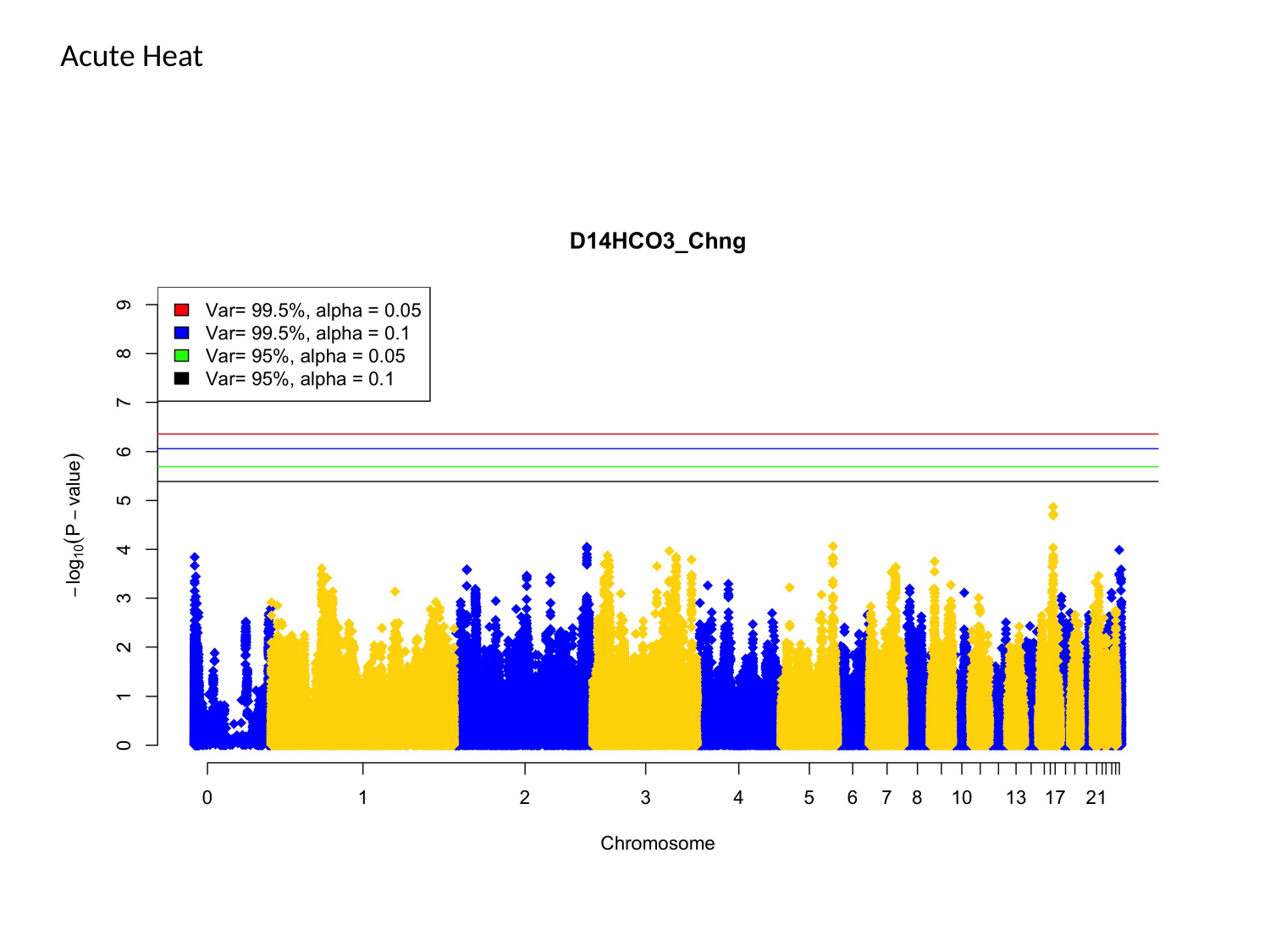

Acute Heat

## Slide 5
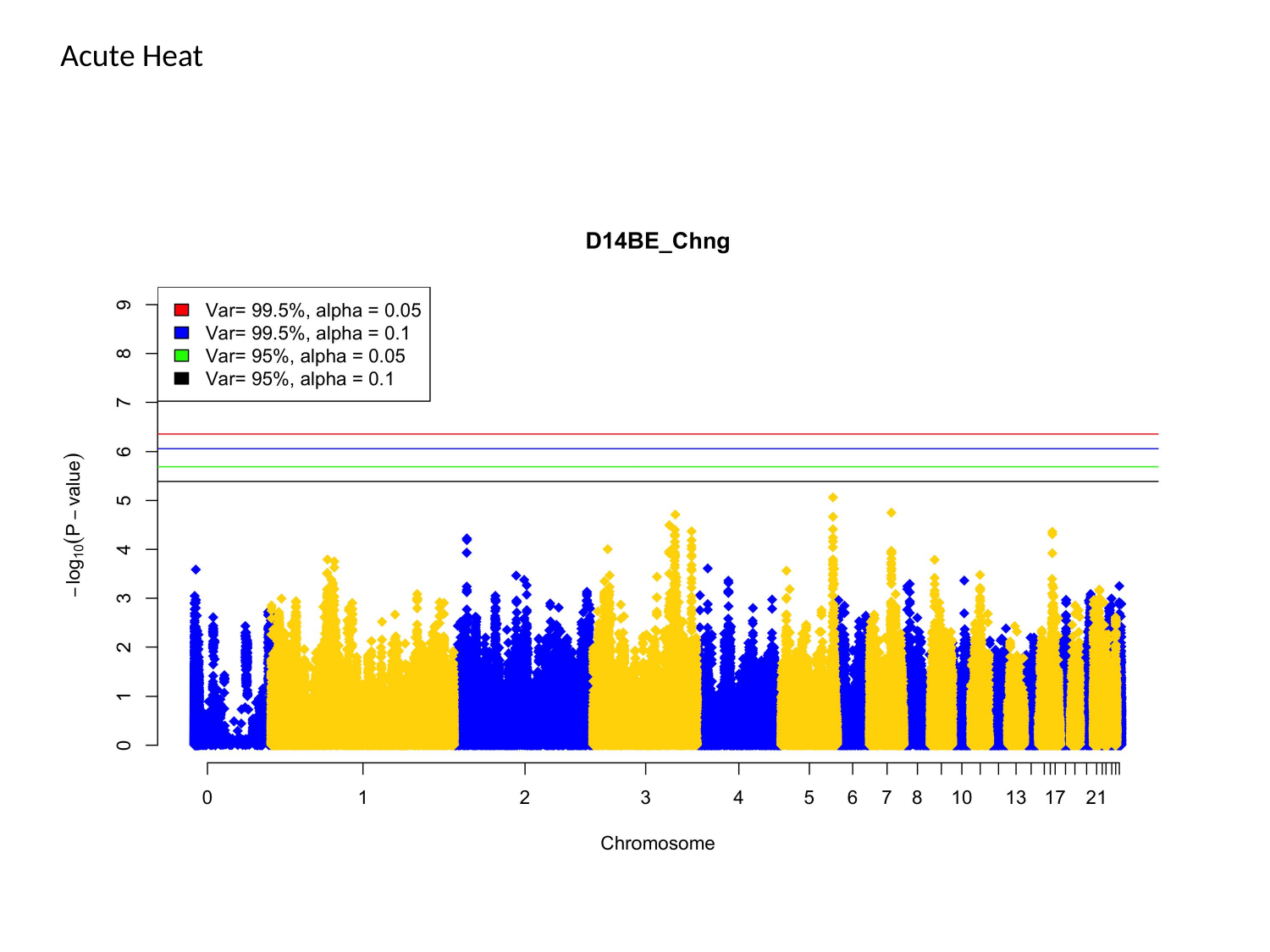

Acute Heat

## Slide 6
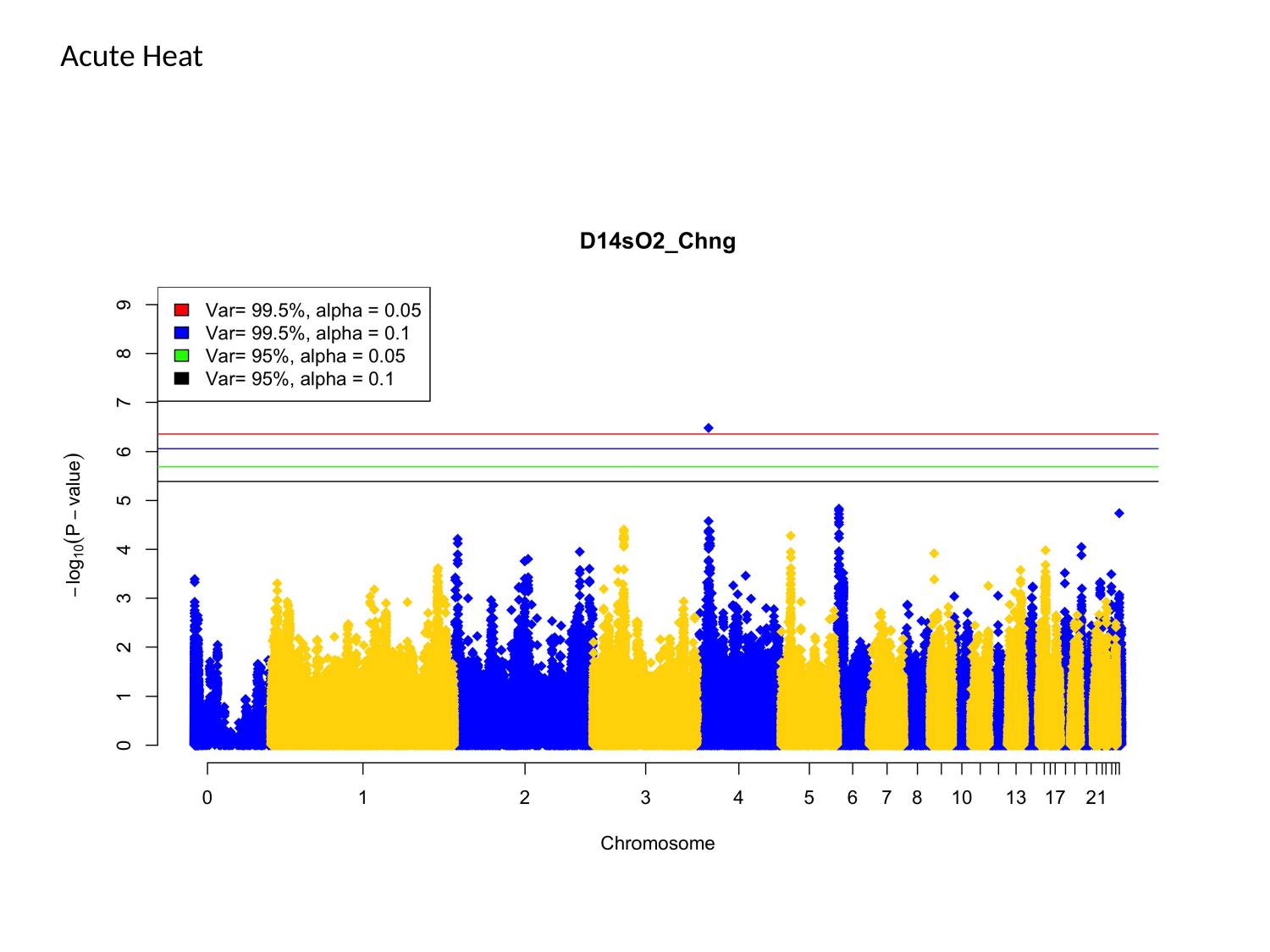

Acute Heat

## Slide 7
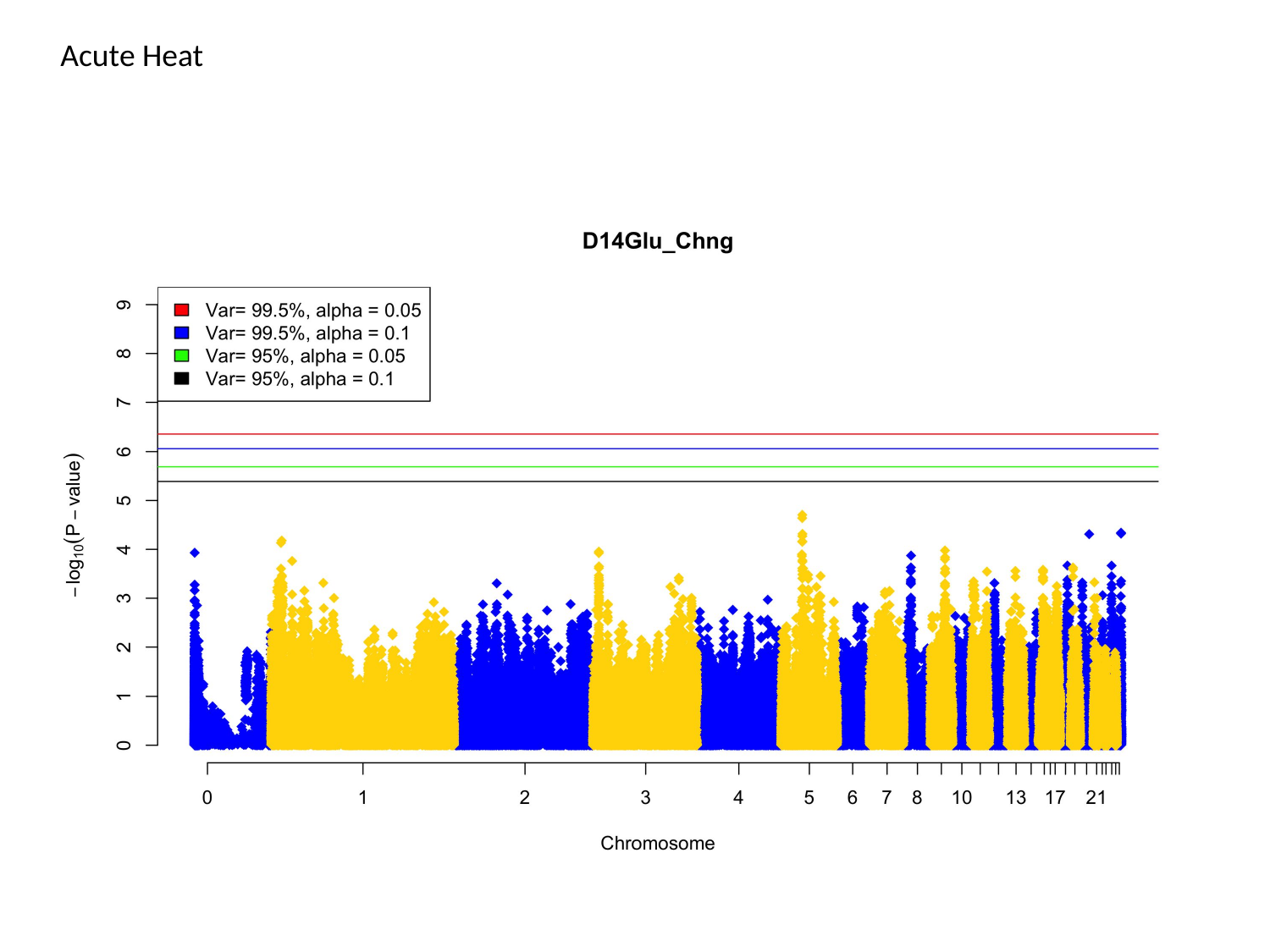

Acute Heat

## Slide 8
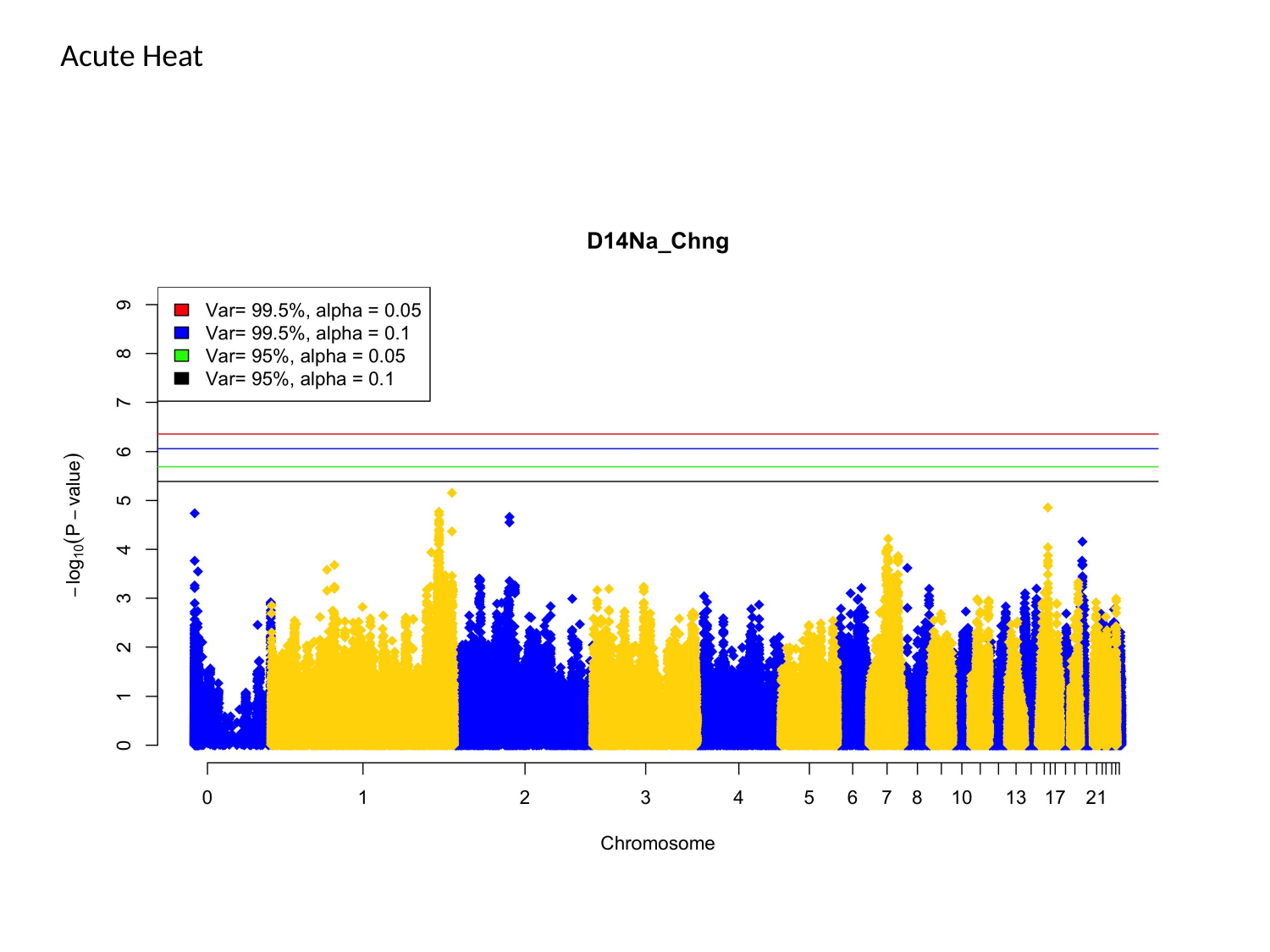

Acute Heat

## Slide 9
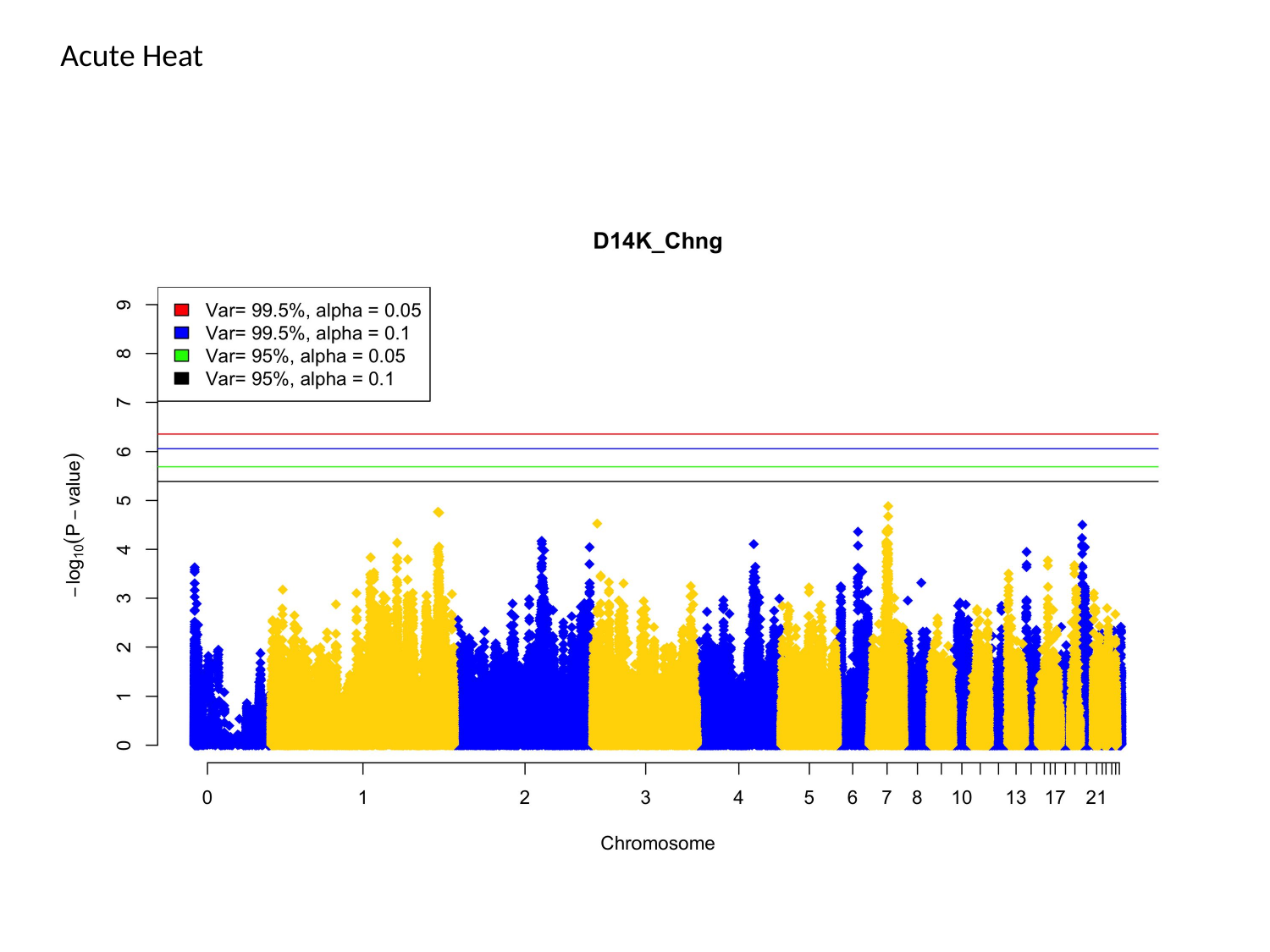

Acute Heat

## Slide 10
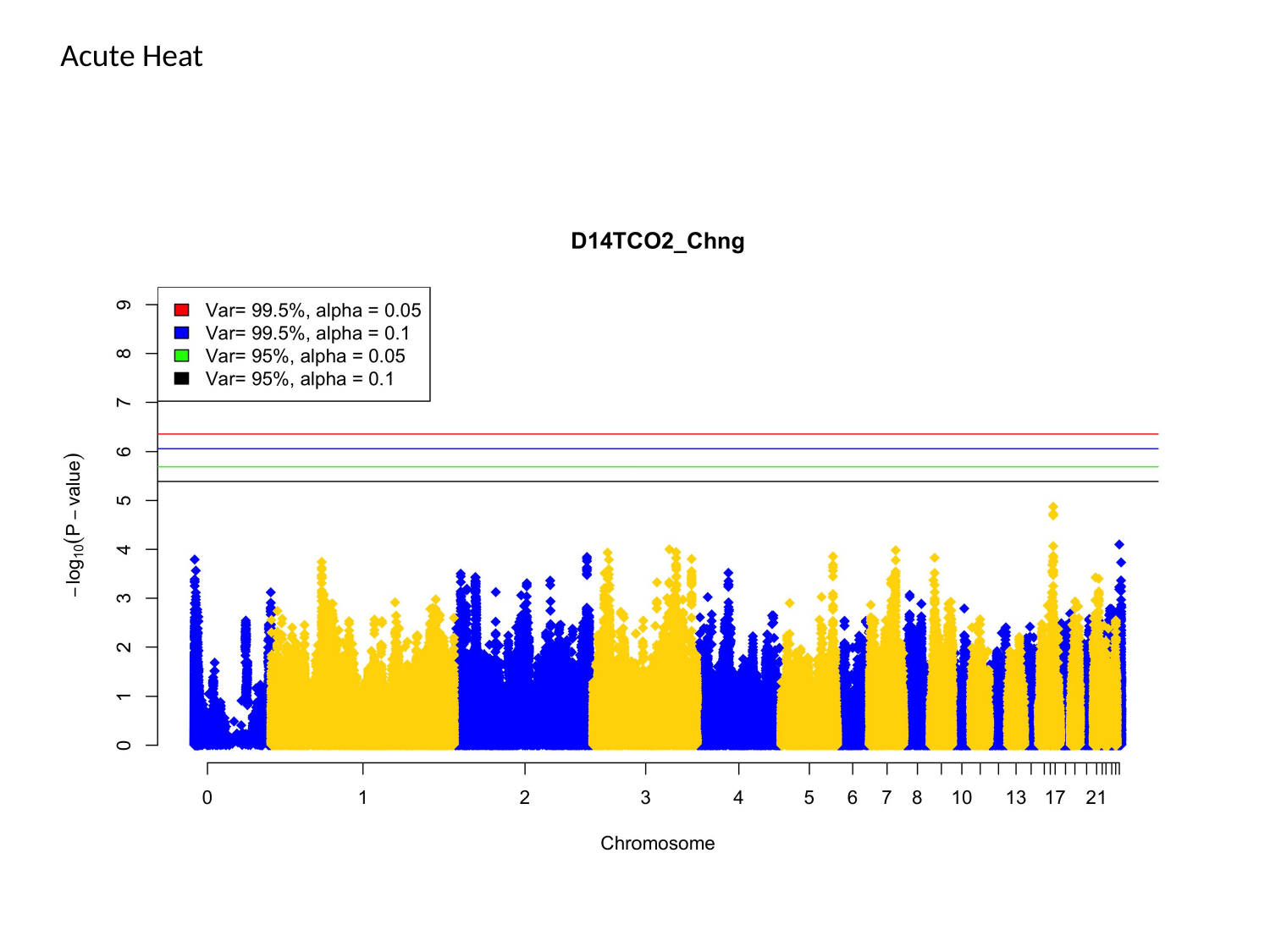

Acute Heat

## Slide 11
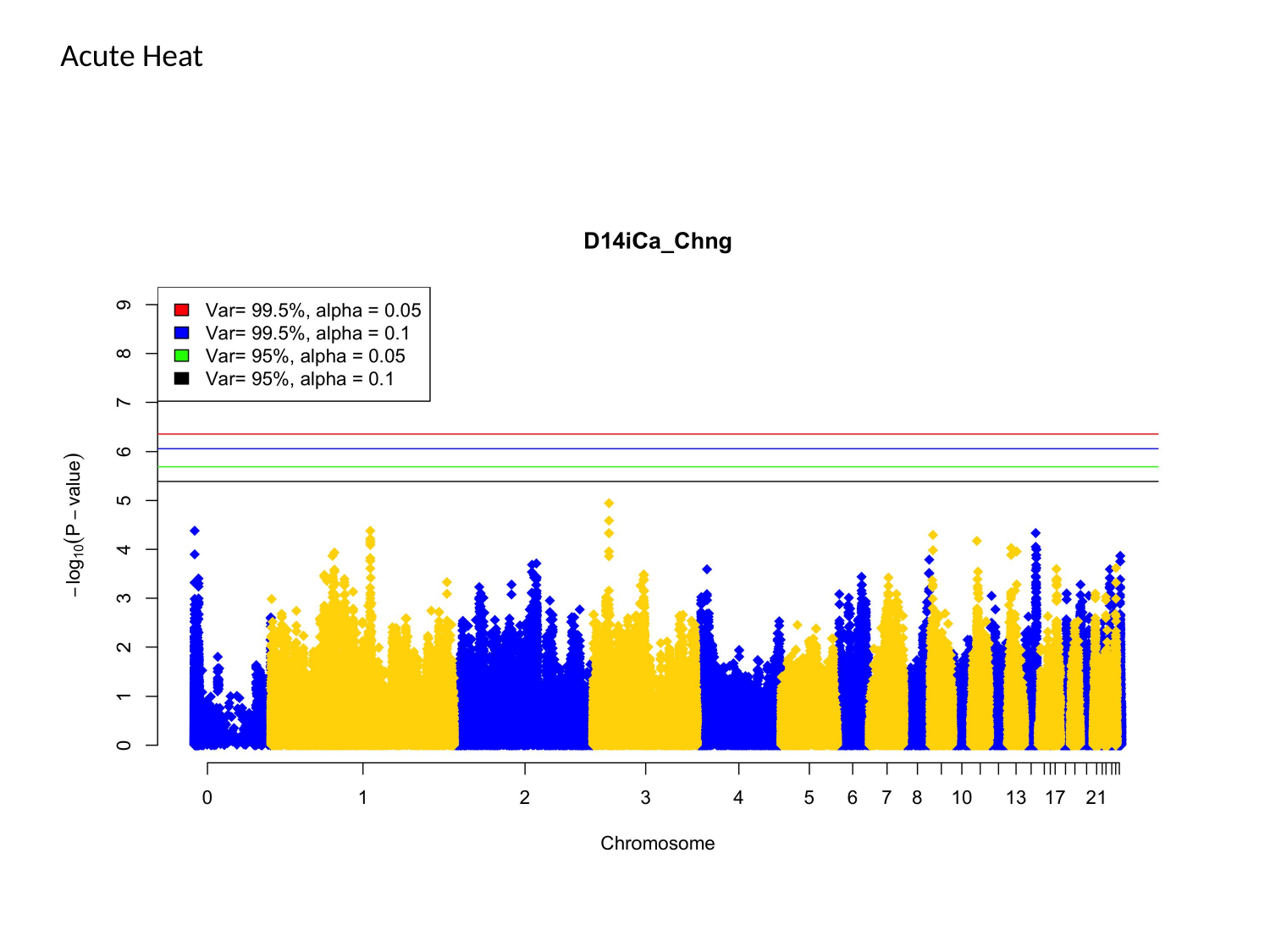

Acute Heat

## Slide 12
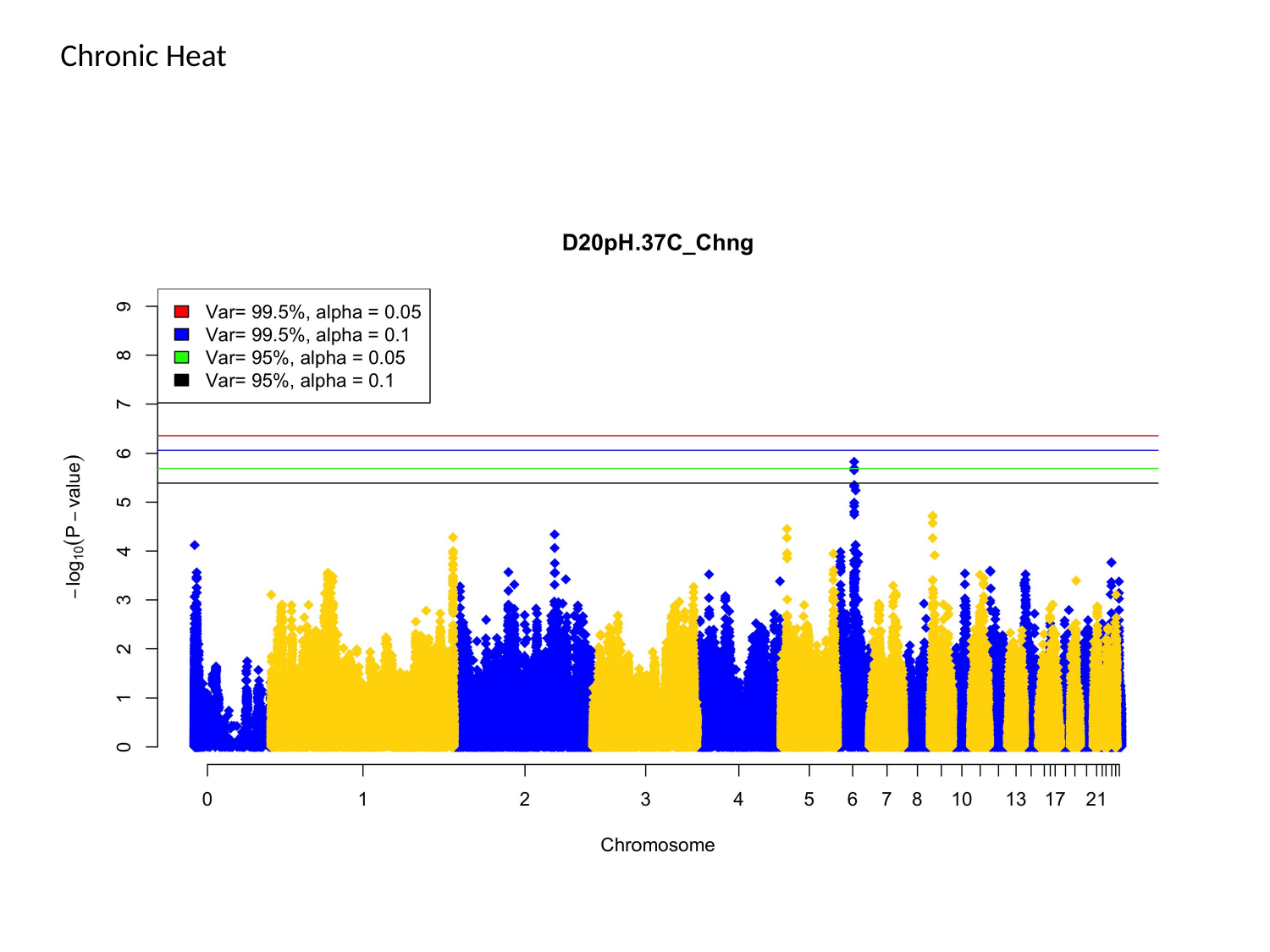

Chronic Heat

## Slide 13
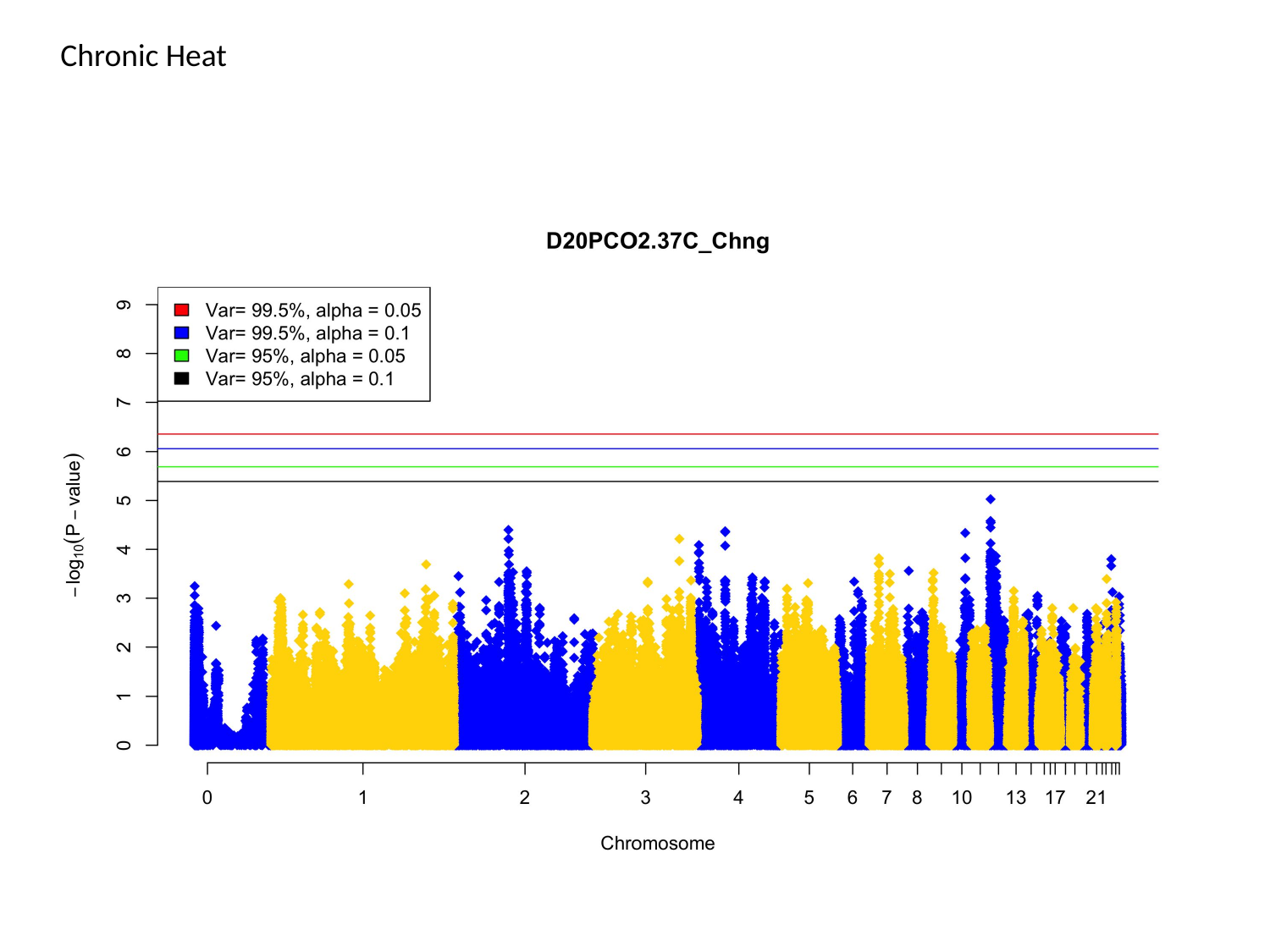

Chronic Heat

## Slide 14
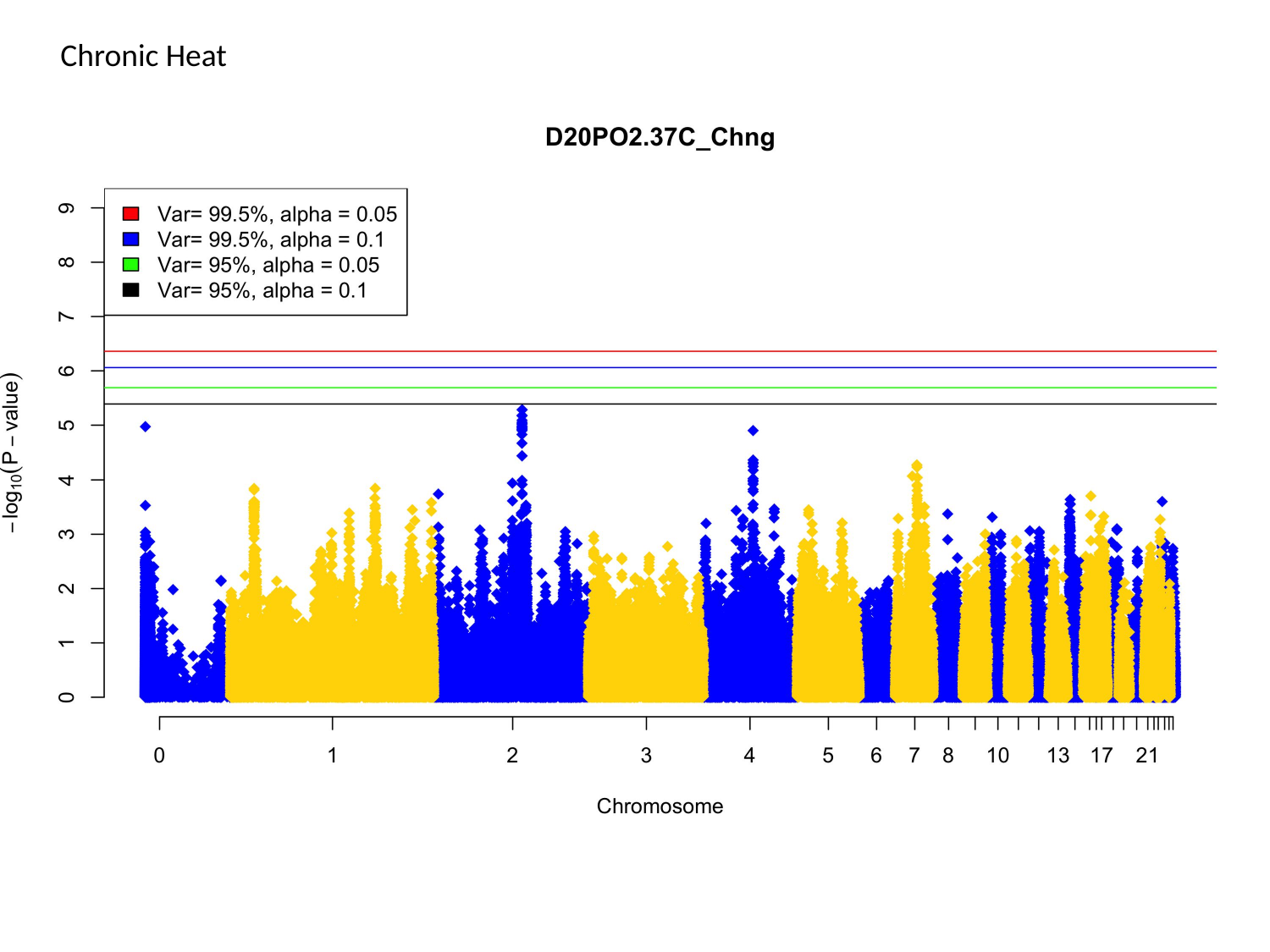

Chronic Heat

## Slide 15
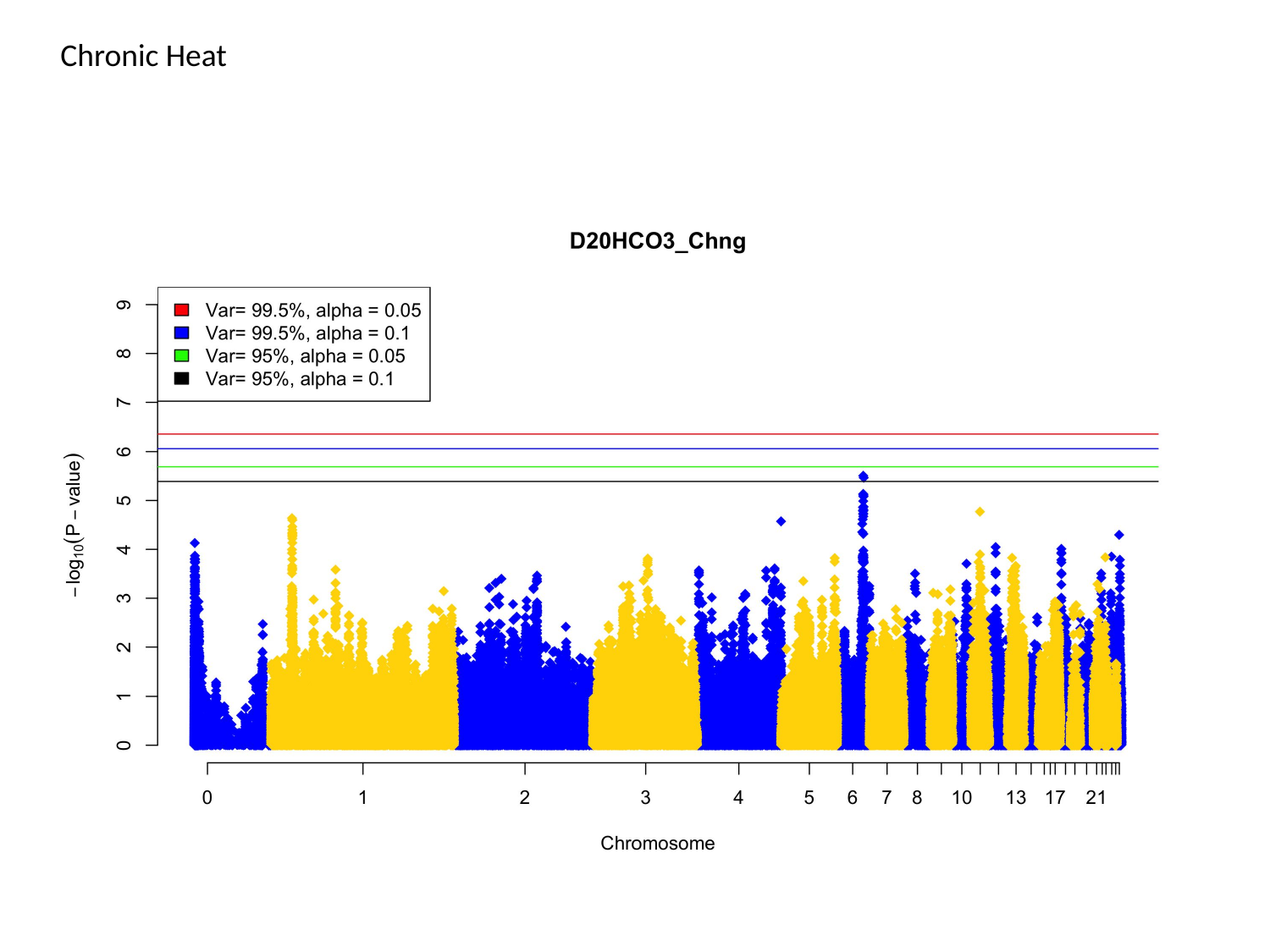

Chronic Heat

## Slide 16
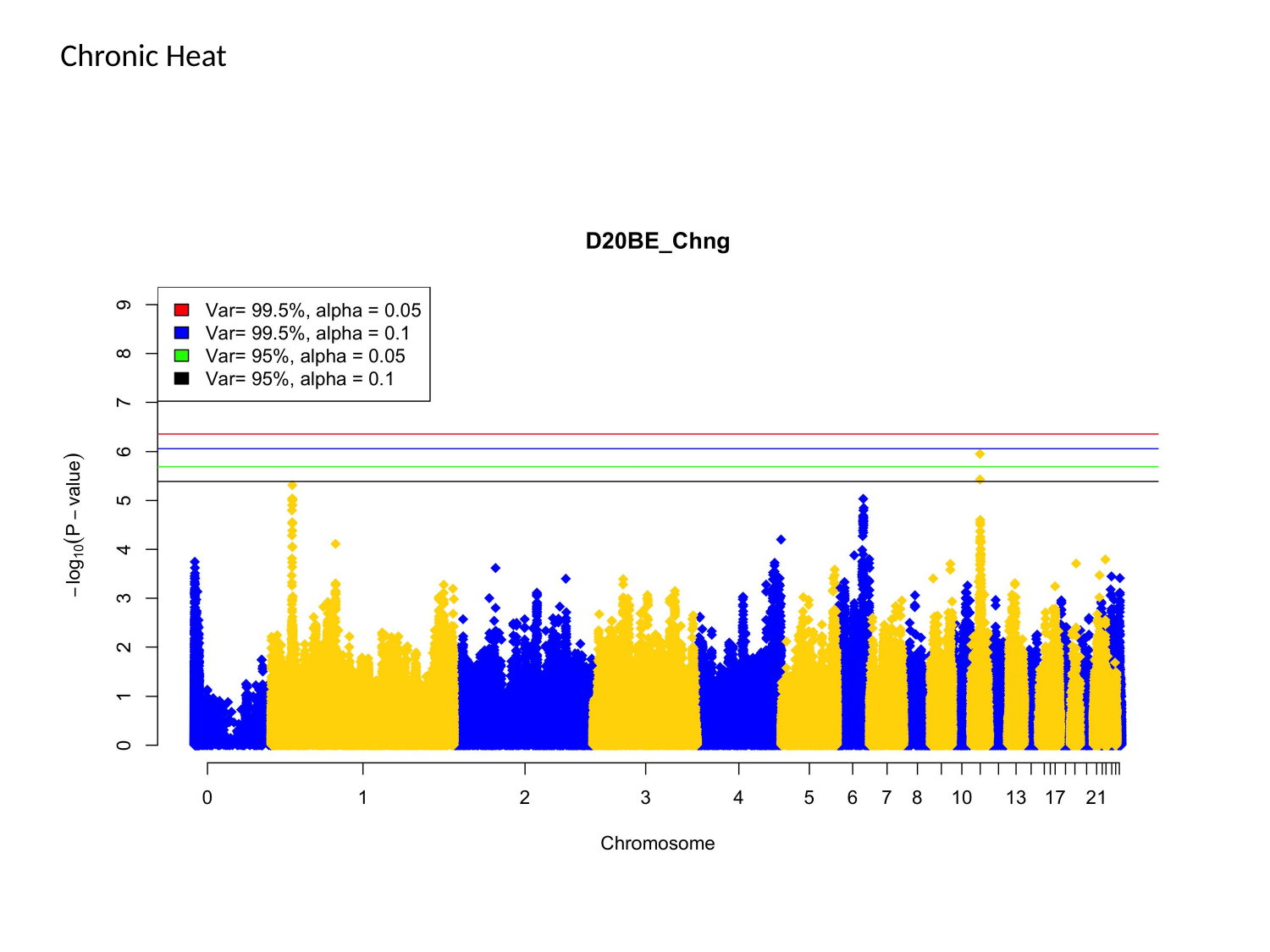

Chronic Heat

## Slide 17
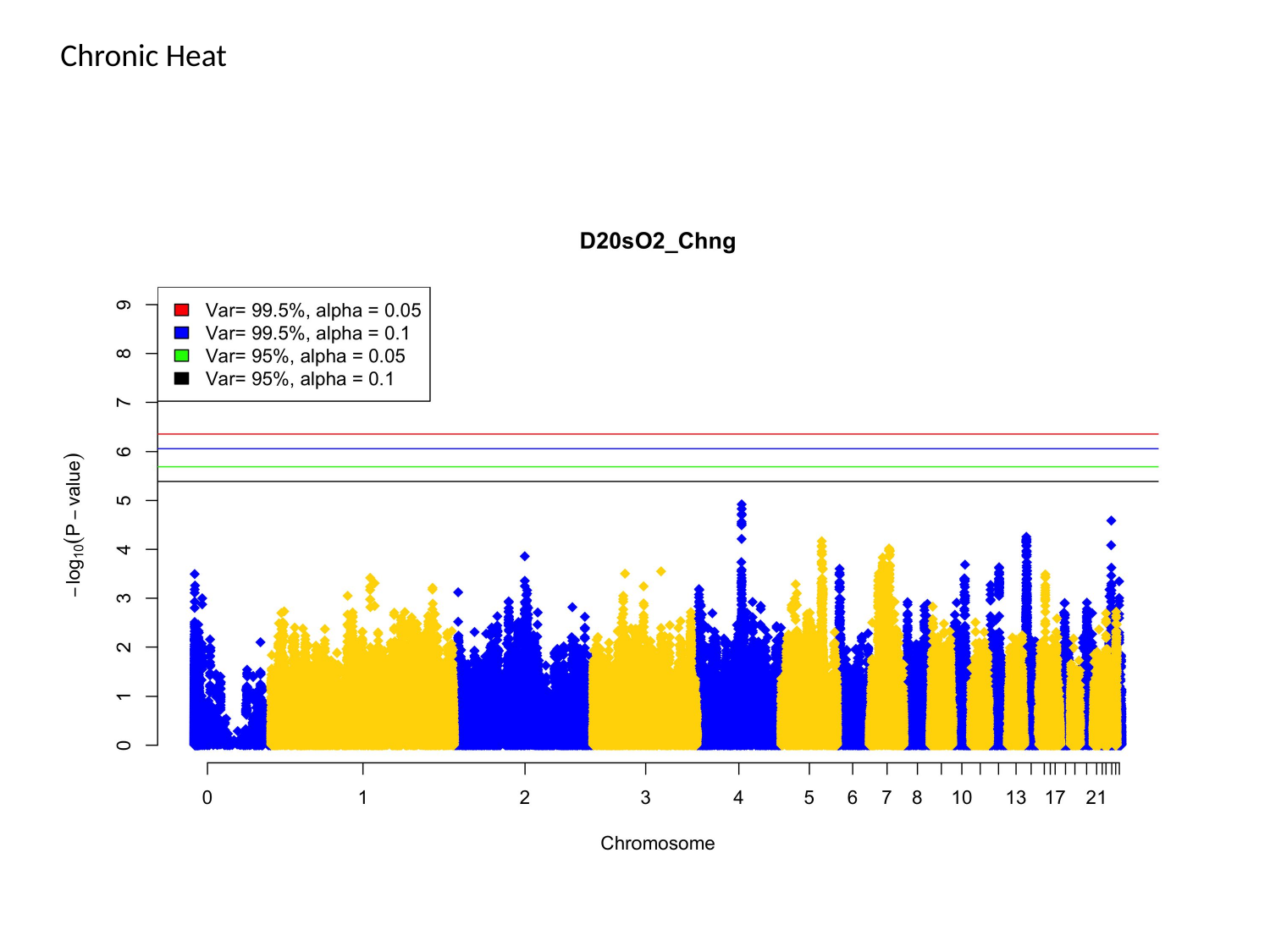

Chronic Heat

## Slide 18
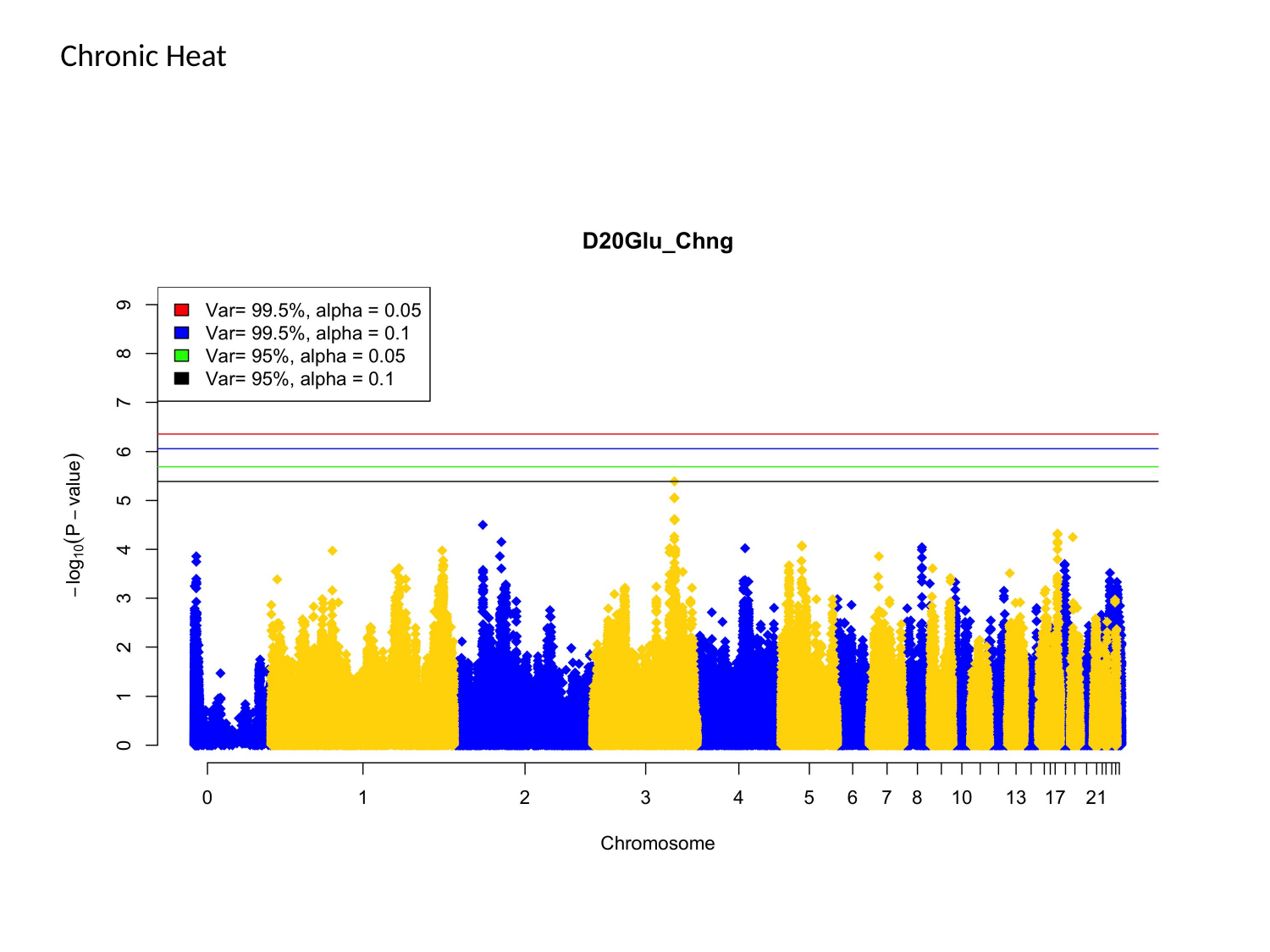

Chronic Heat

## Slide 19
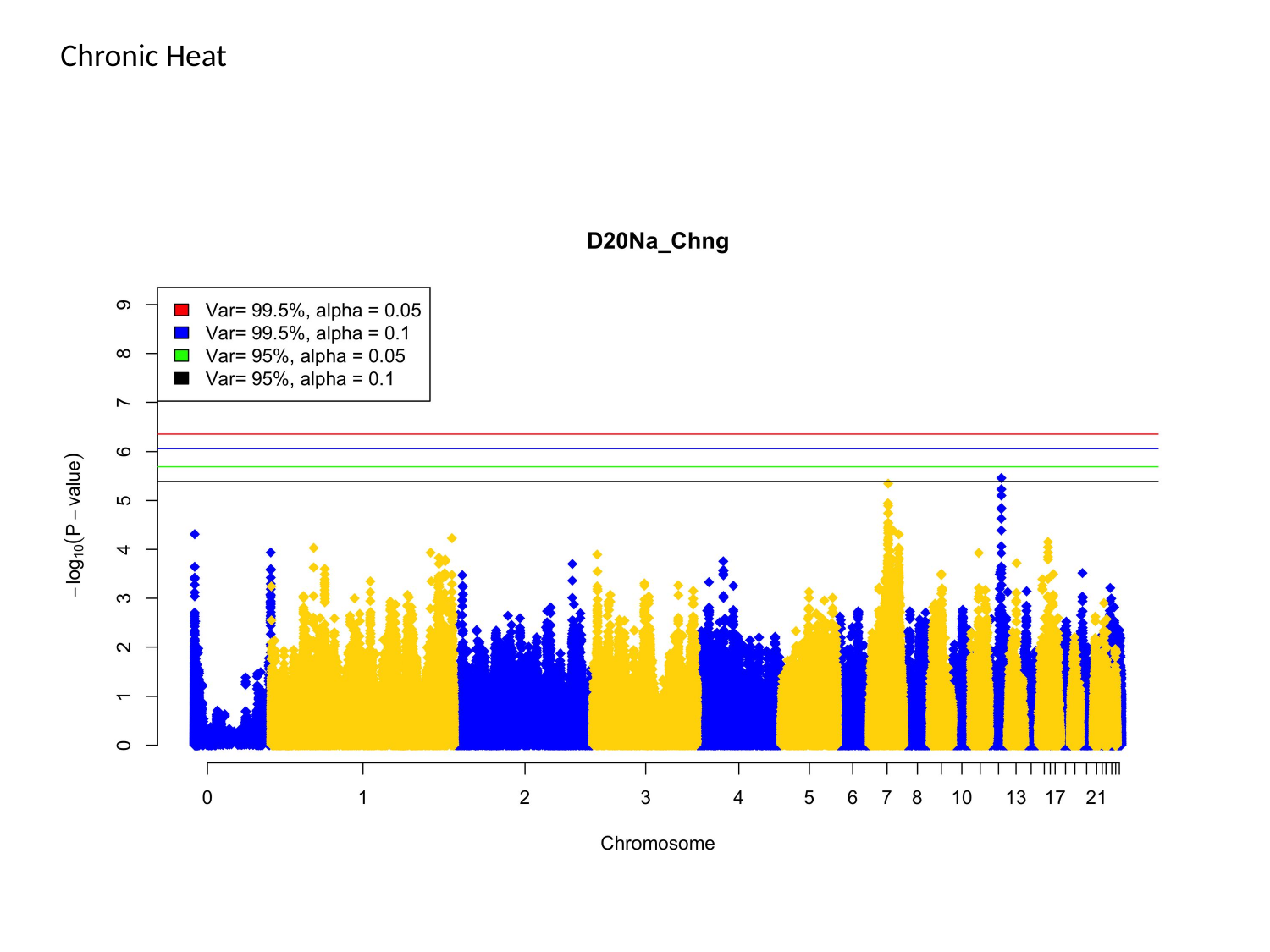

Chronic Heat

## Slide 20
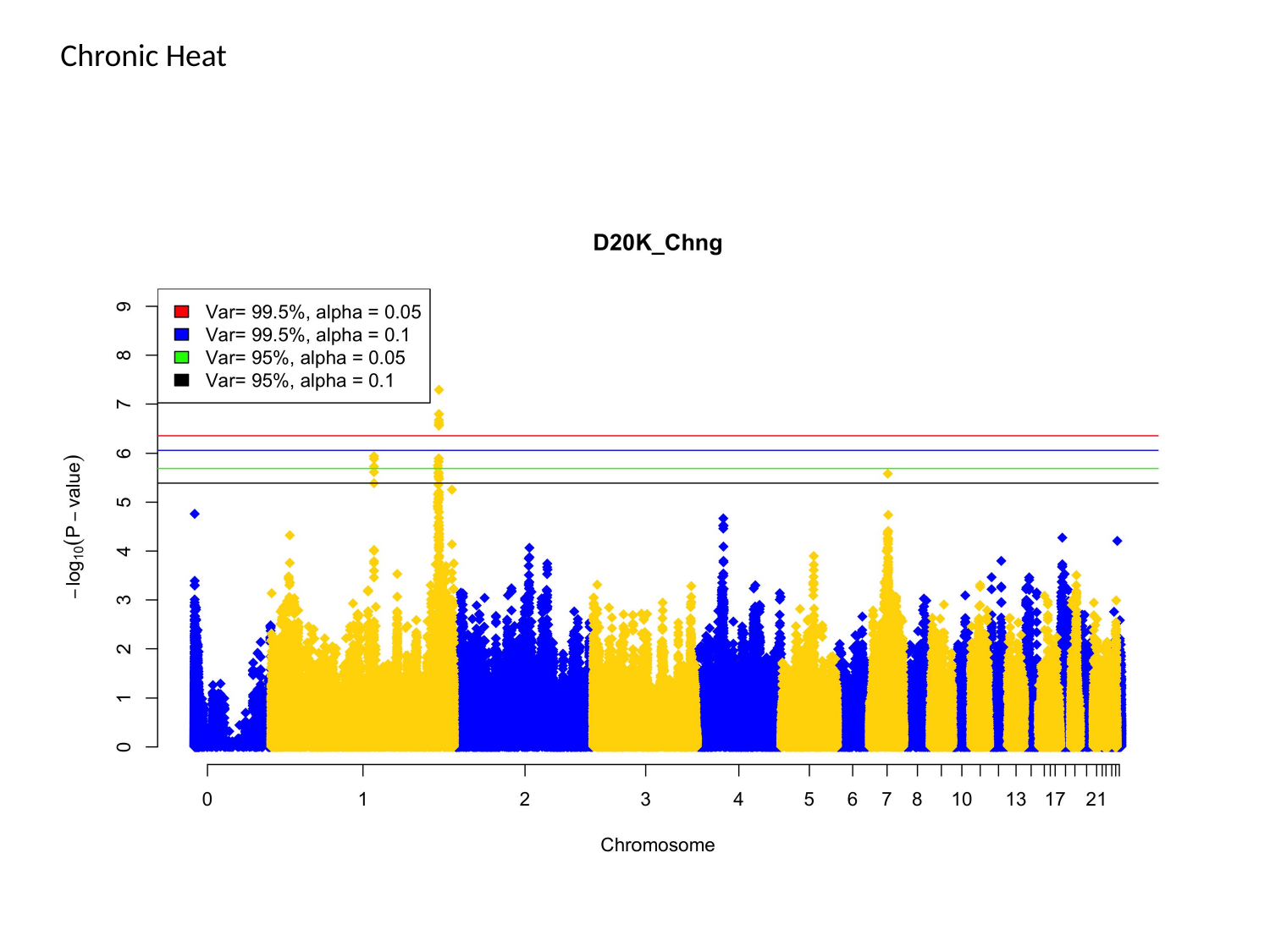

Chronic Heat

## Slide 21
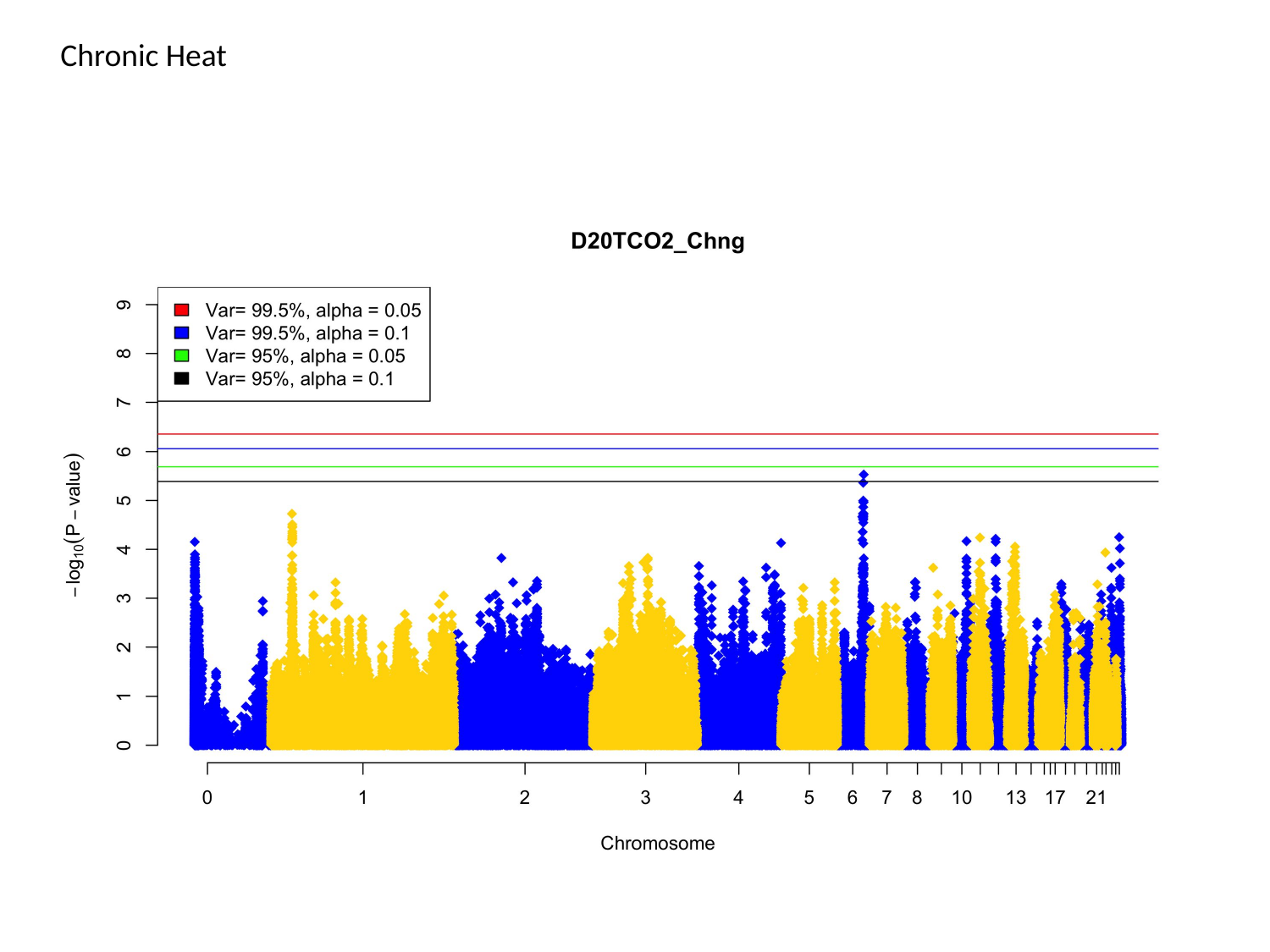

Chronic Heat

## Slide 22
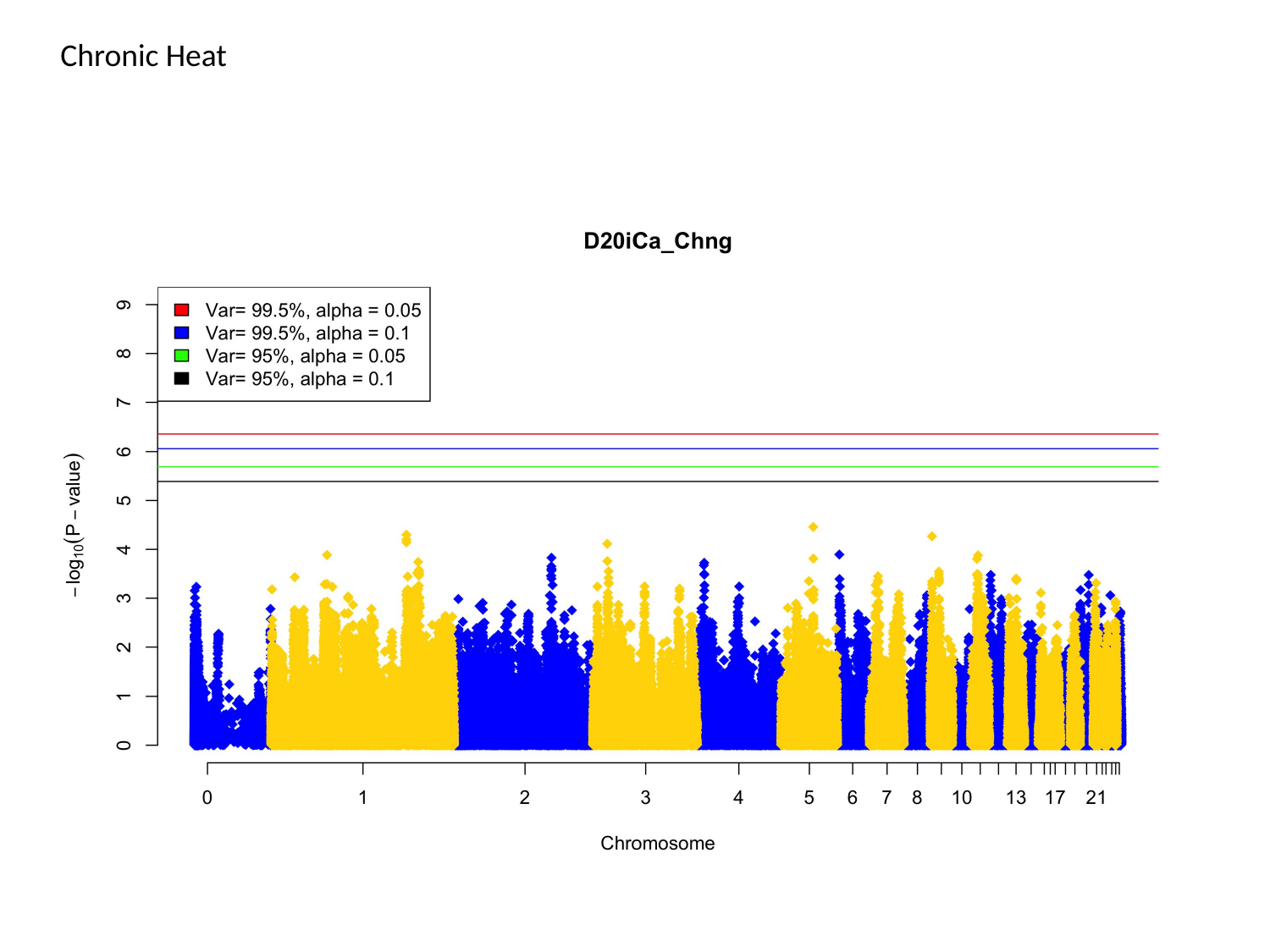

Chronic Heat

## Slide 23
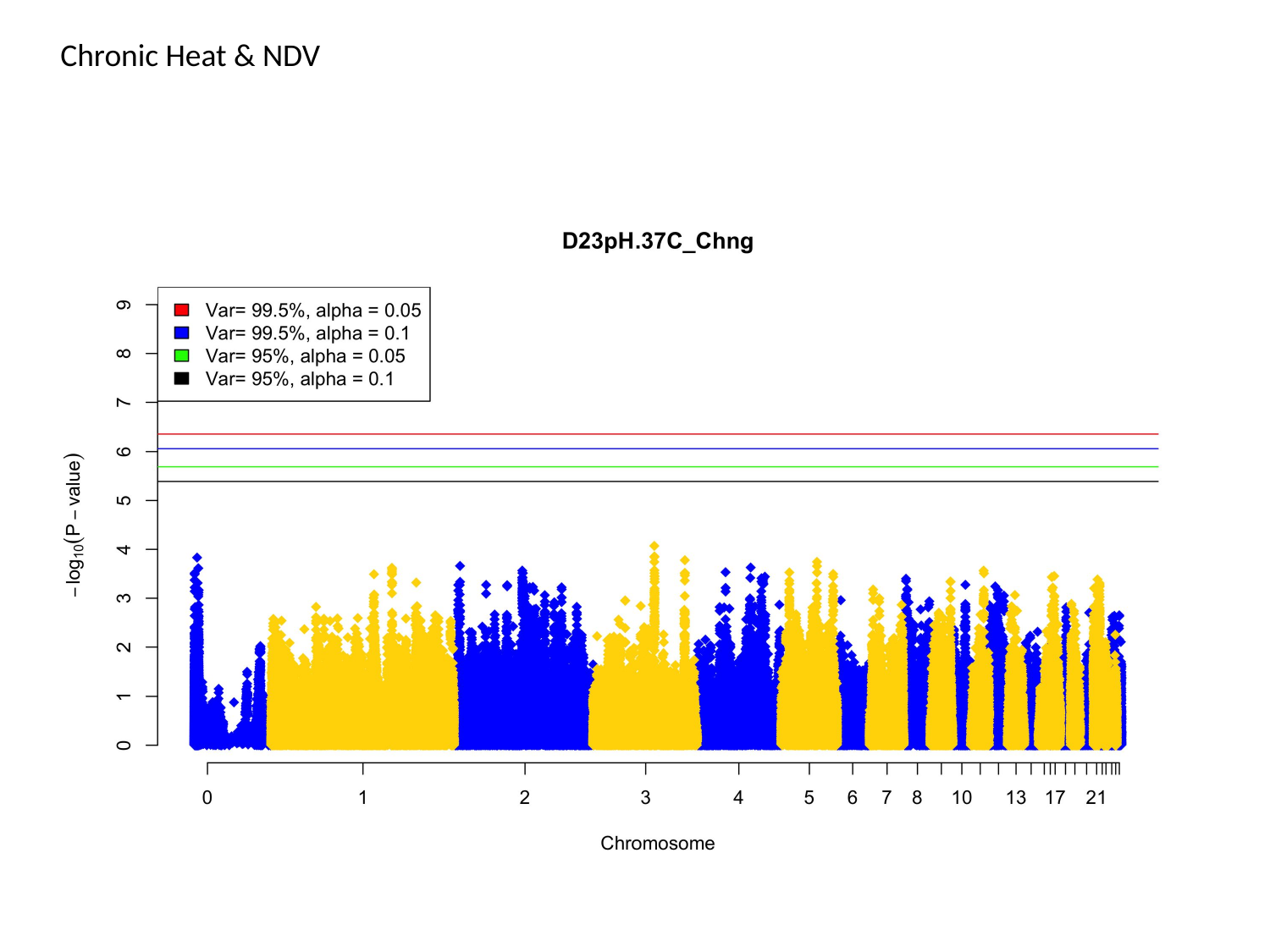

Chronic Heat & NDV

## Slide 24
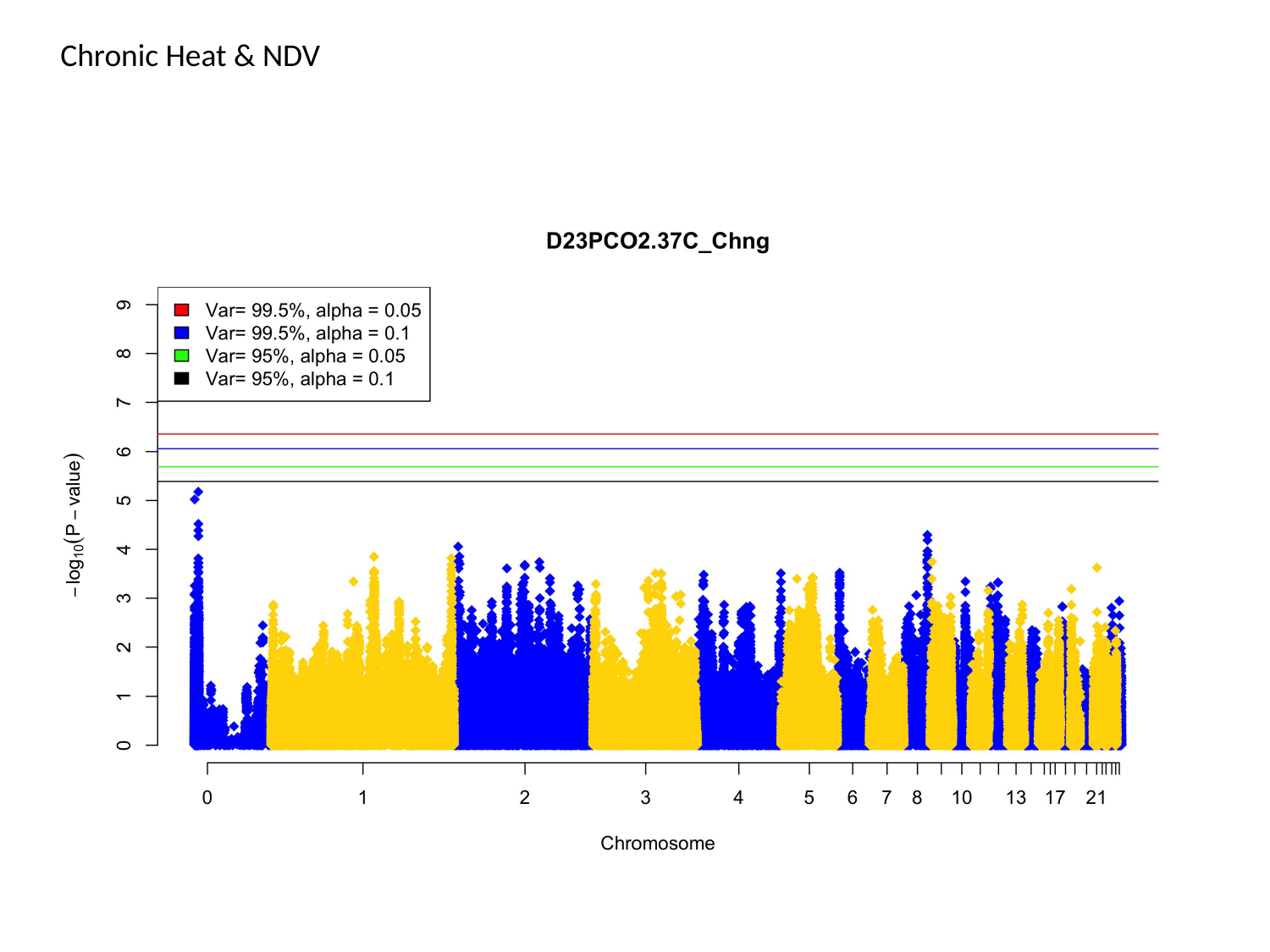

Chronic Heat & NDV

## Slide 25
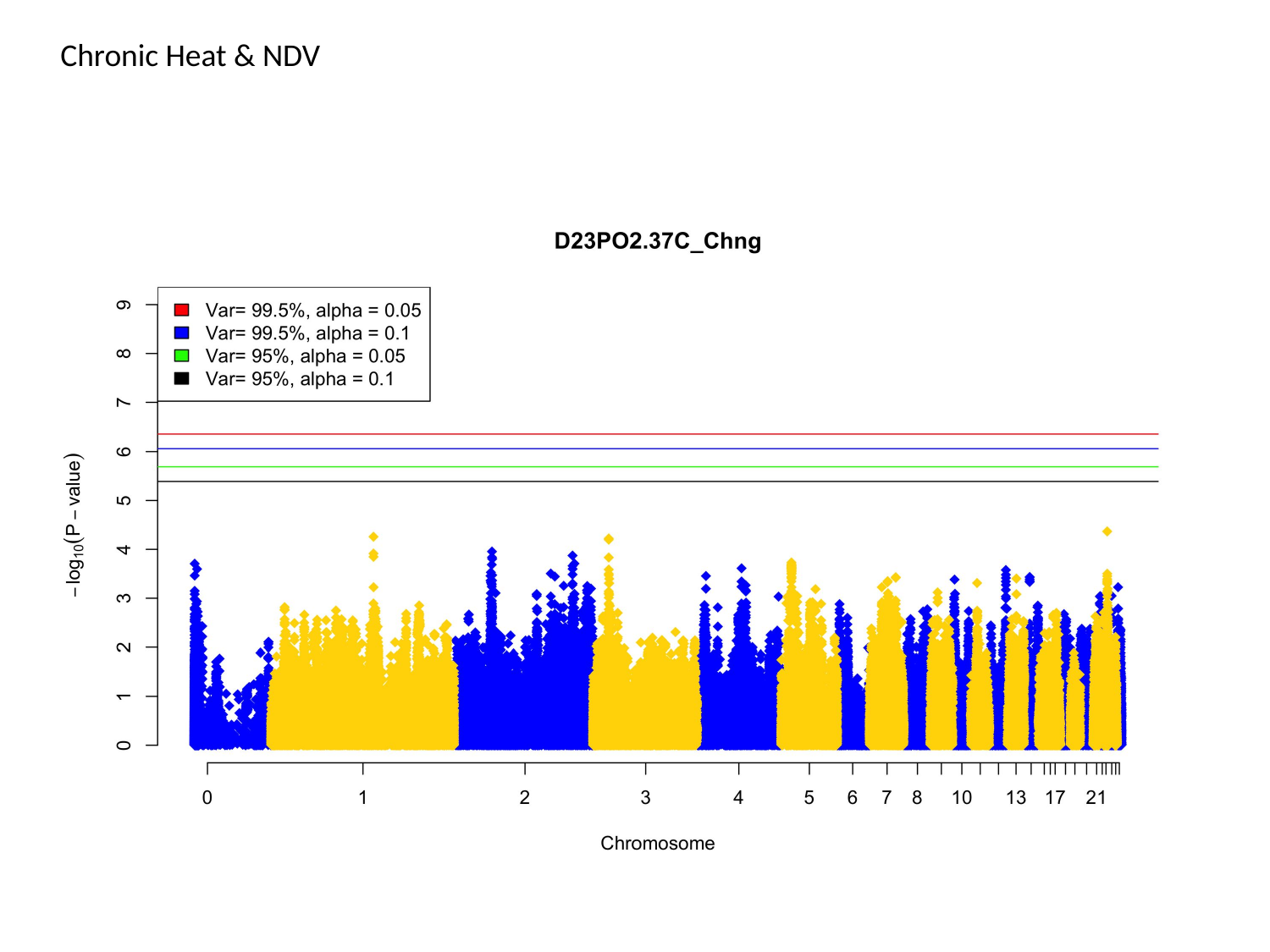

Chronic Heat & NDV

## Slide 26
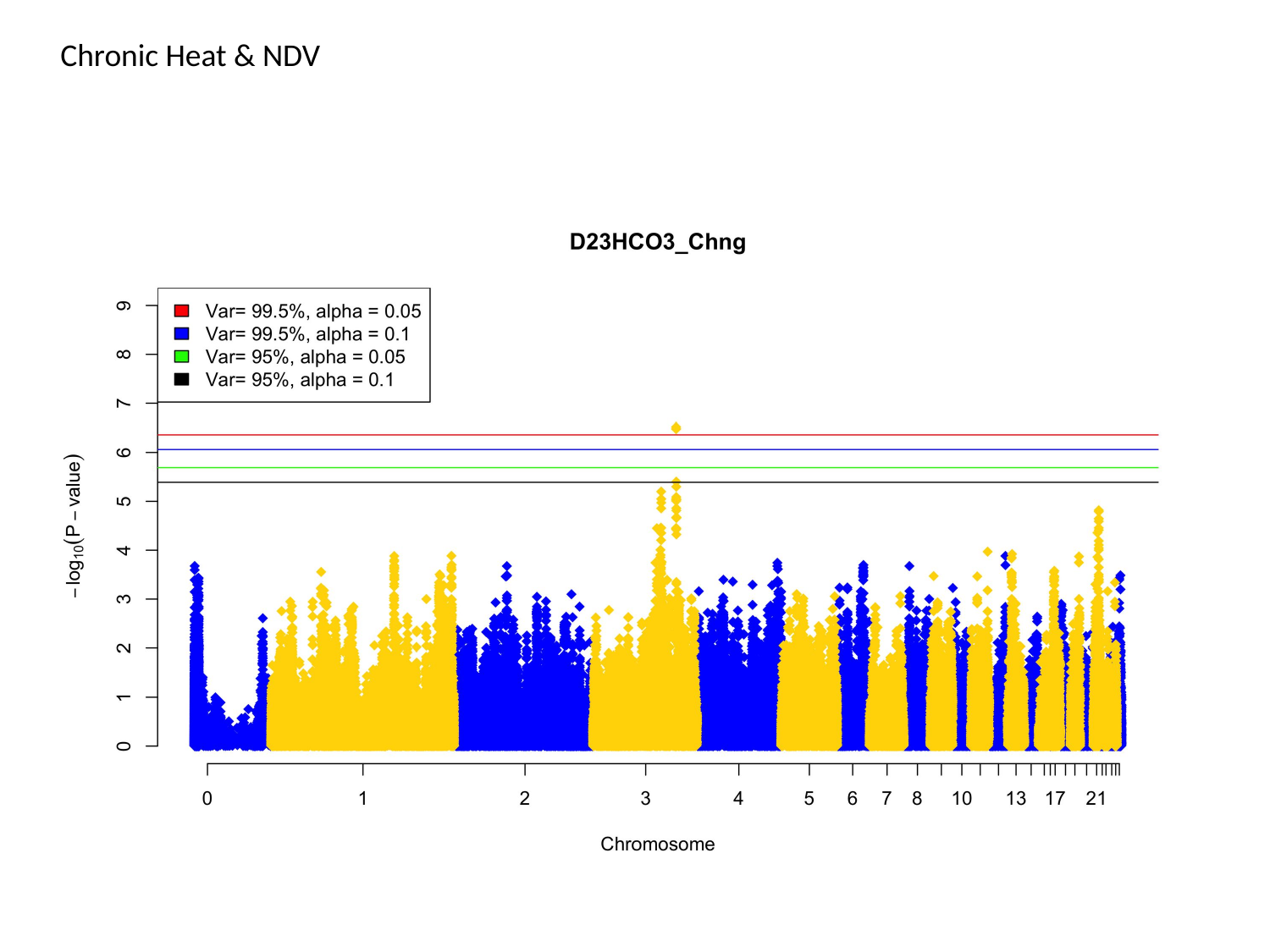

Chronic Heat & NDV

## Slide 27
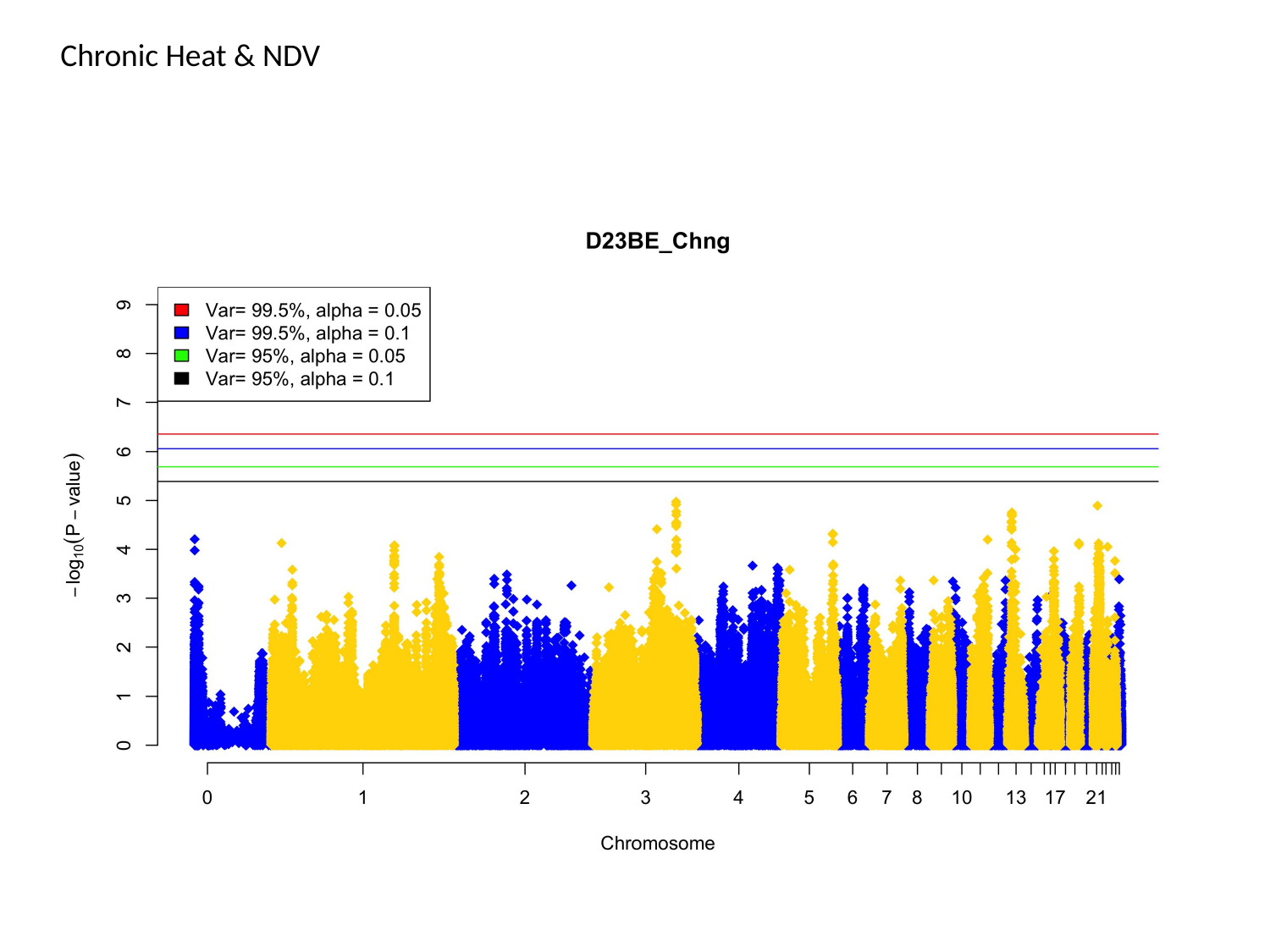

Chronic Heat & NDV

## Slide 28
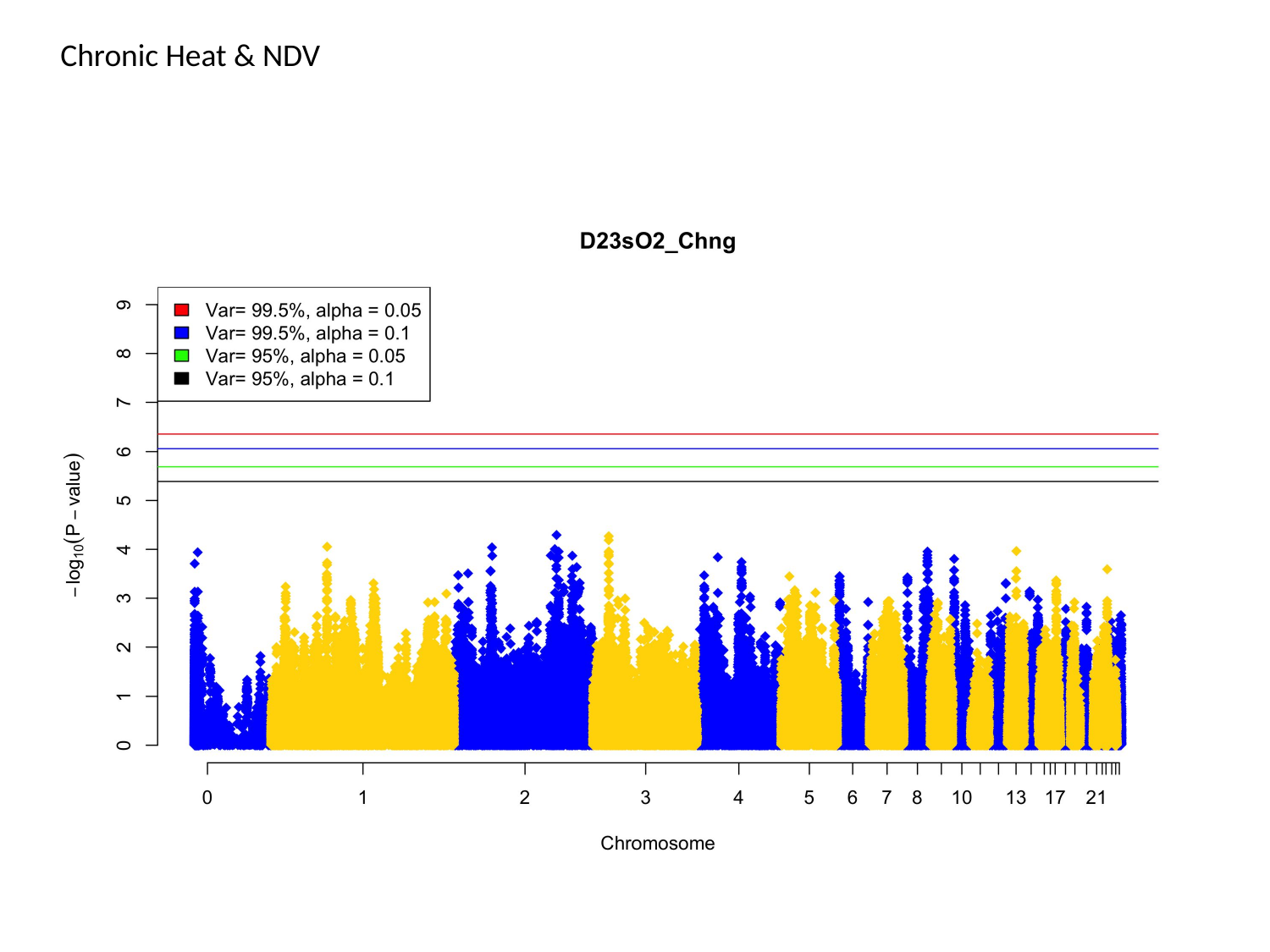

Chronic Heat & NDV

## Slide 29
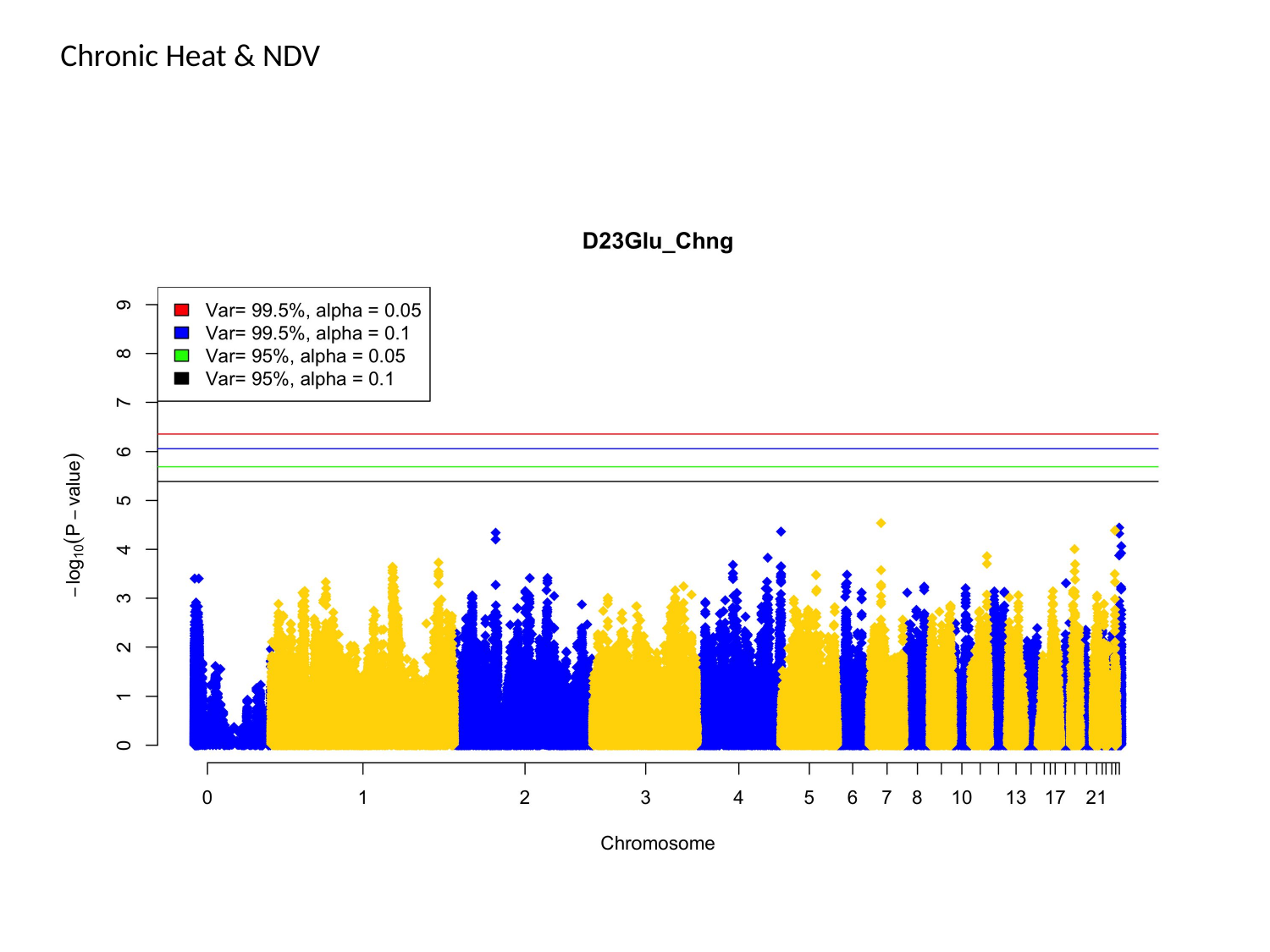

Chronic Heat & NDV

## Slide 30
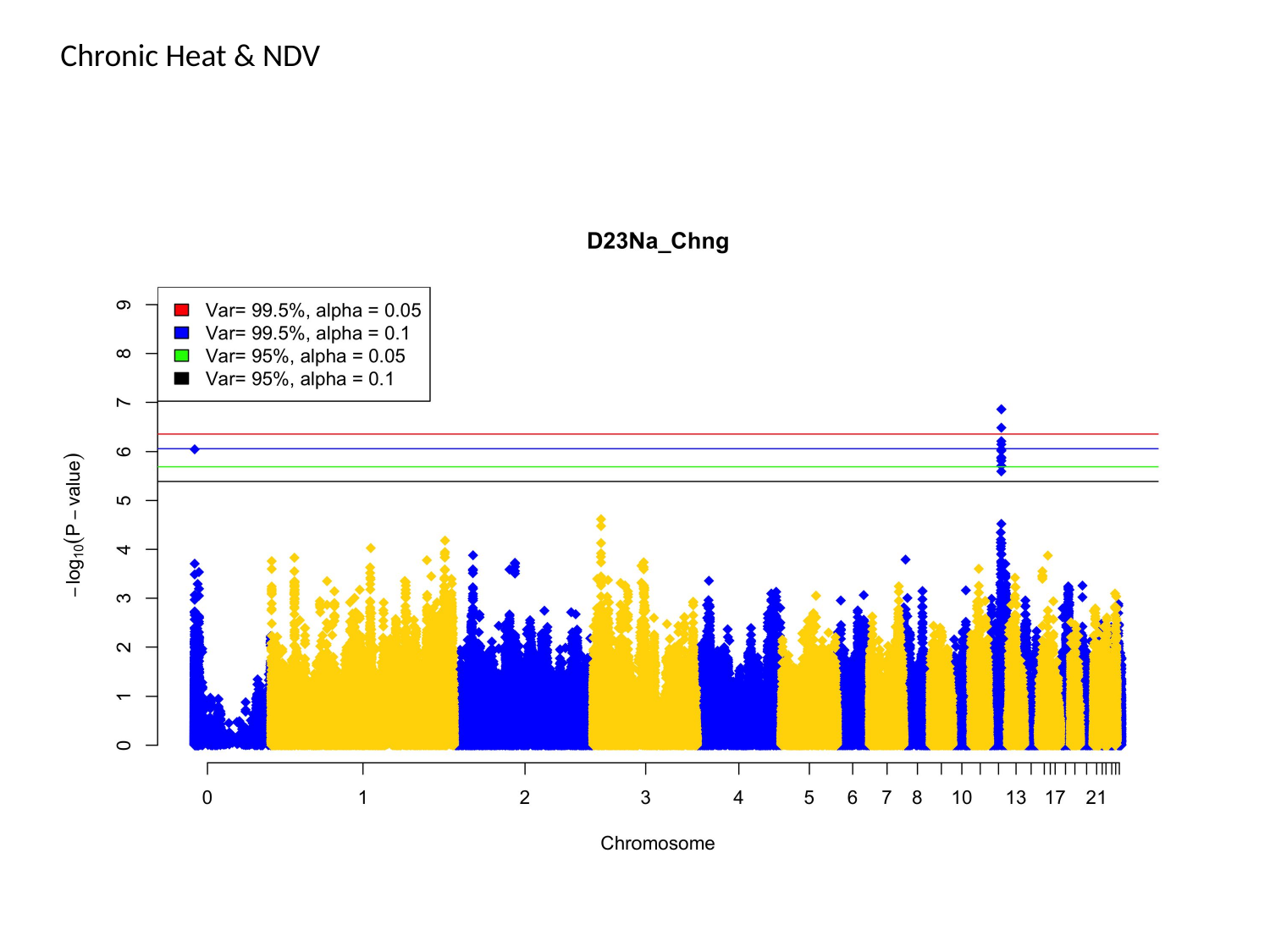

Chronic Heat & NDV

## Slide 31
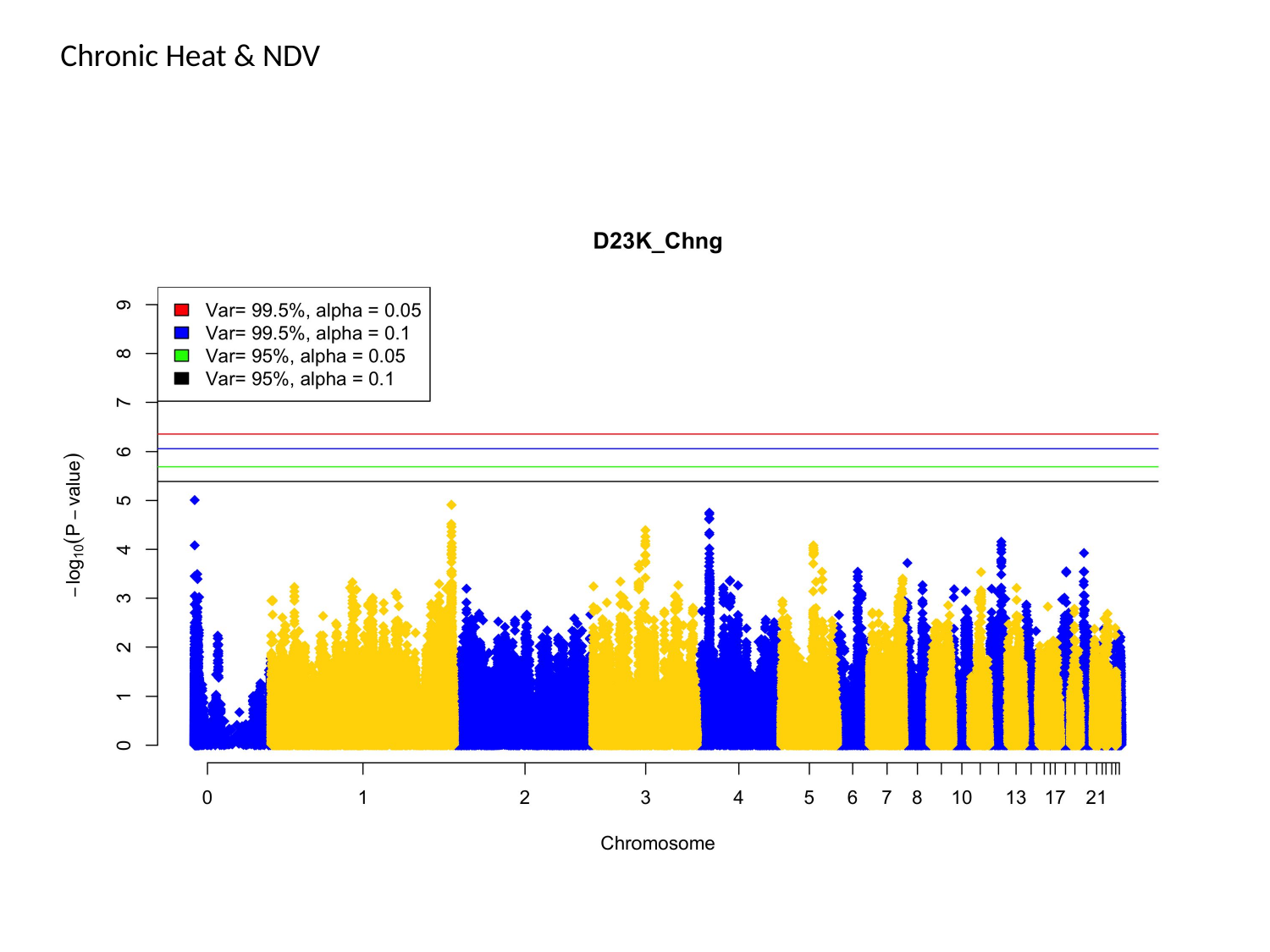

Chronic Heat & NDV

## Slide 32
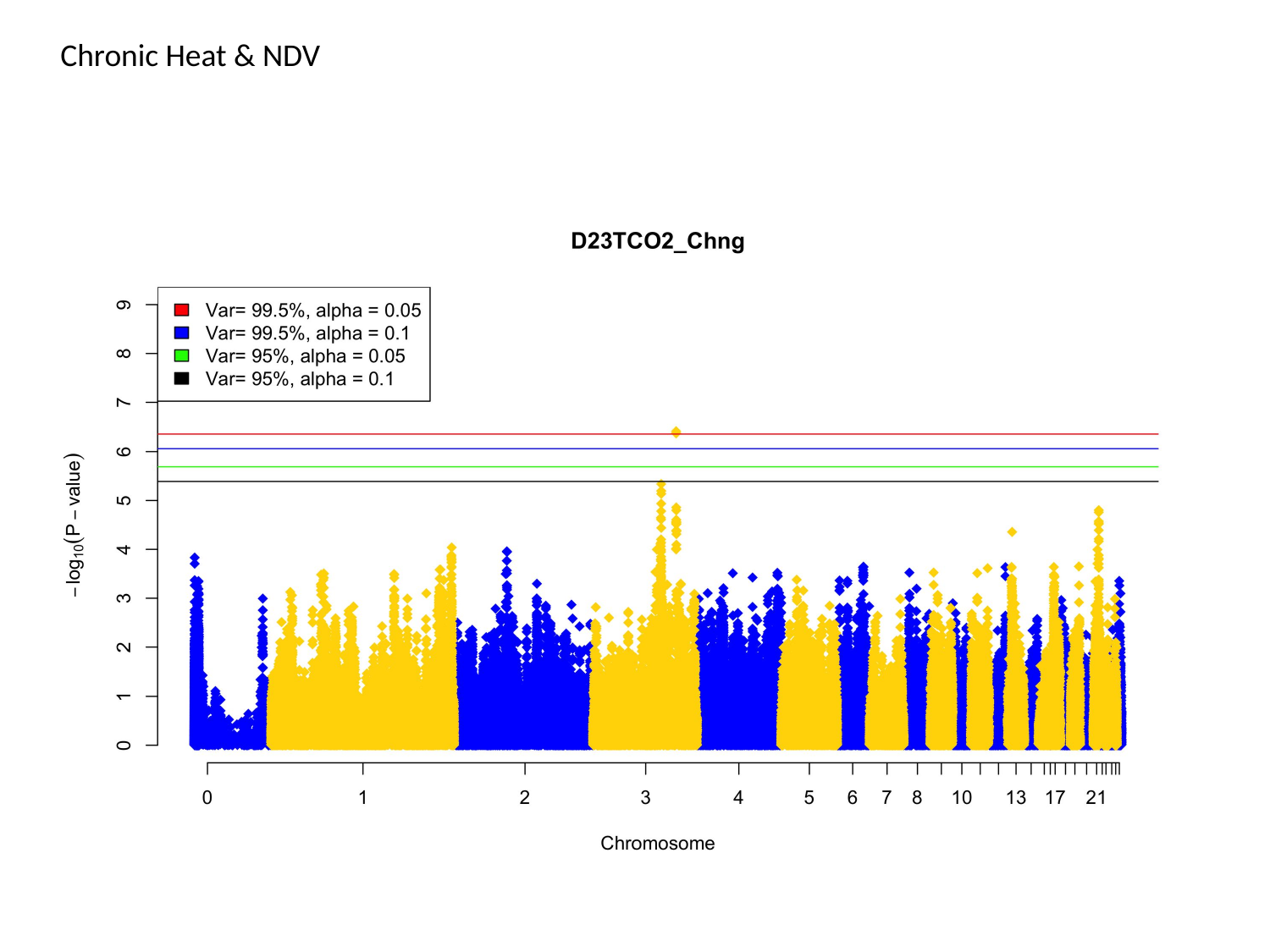

Chronic Heat & NDV

## Slide 33
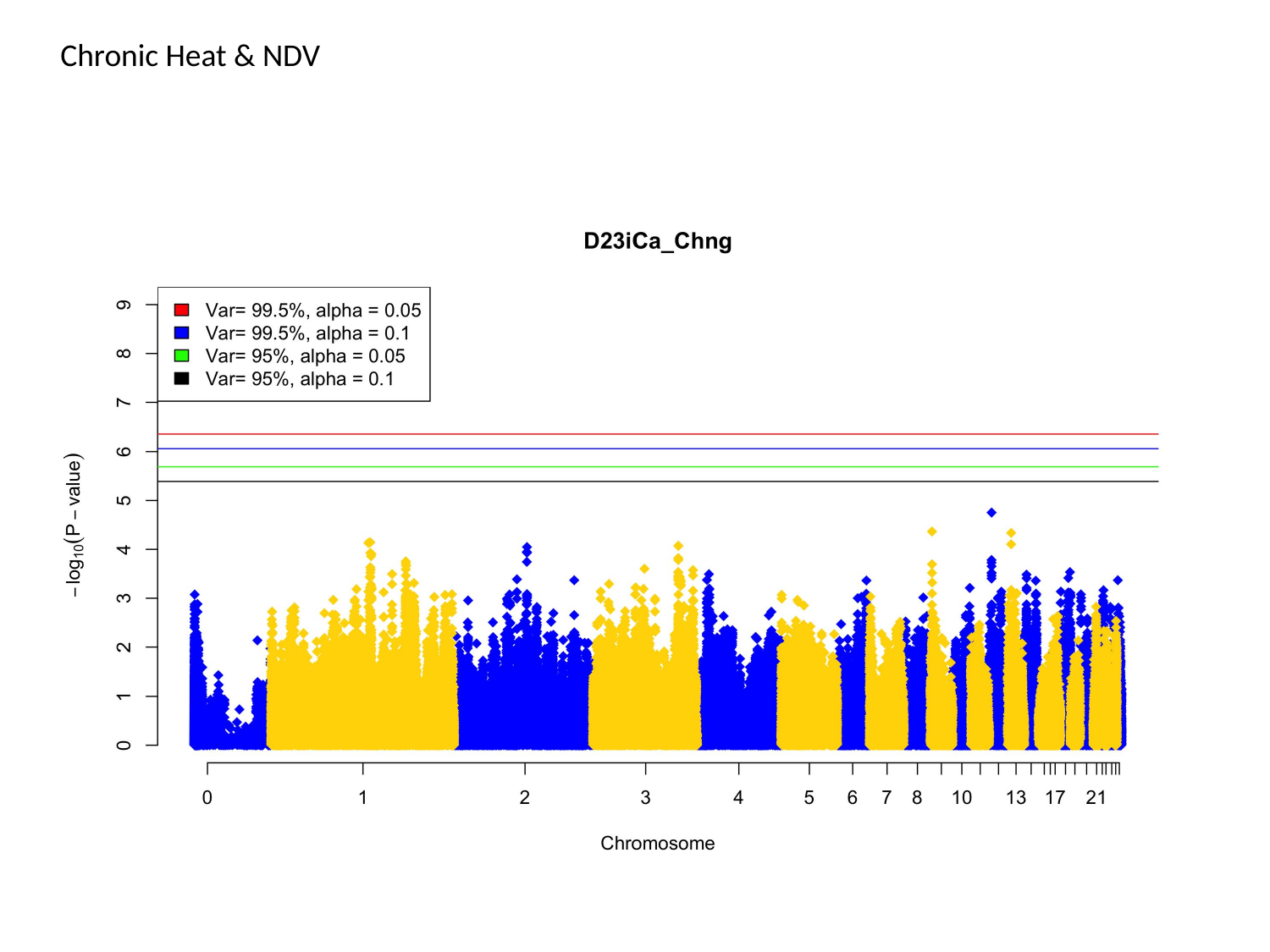

Chronic Heat & NDV
